# Nonadiabatic Absorption Spectra and Ultrafast Dynamics of DNA and RNA Photoexcited Nucleobases

**DOI:** 10.3390/molecules26061743

**Published:** 2021-03-20

**Authors:** James A. Green, Martha Yaghoubi Jouybari, Daniel Aranda, Roberto Improta, Fabrizio Santoro

**Affiliations:** 1 CNR—Consiglio Nazionale delle Ricerche, Istituto di Biostrutture e Bioimmagini (IBB-CNR), Via Mezzocannone 16, I-80136 Napoli, Italy; james.green@ibb.cnr.it; 2 CNR—Consiglio Nazionale Delle Ricerche, Istituto di Chimica dei Composti Organo Metallici (ICCOM-CNR), SS di Pisa, Area Della Ricerca, Via G. Moruzzi 1, I-56124 Pisa, Italy; martha.yaghoubi@pi.iccom.cnr.it (M.Y.J.); aranda@uma.es (D.A.)

**Keywords:** photoinduced processes, nonadiabatic interactions, quantum dynamics, vibronic spectra, nucleobases

## Abstract

We have recently proposed a protocol for Quantum Dynamics (QD) calculations, which is based on a parameterisation of Linear Vibronic Coupling (LVC) Hamiltonians with Time Dependent (TD) Density Functional Theory (TD-DFT), and exploits the latest developments in multiconfigurational TD-Hartree methods for an effective wave packet propagation. In this contribution we explore the potentialities of this approach to compute nonadiabatic vibronic spectra and ultrafast dynamics, by applying it to the five nucleobases present in DNA and RNA. For all of them we computed the absorption spectra and the dynamics of ultrafast internal conversion (100 fs timescale), fully coupling the first 2–3 bright states and all the close by dark states, for a total of 6–9 states, and including all the normal coordinates. We adopted two different functionals, CAM-B3LYP and PBE0, and tested the effect of the basis set. Computed spectra are in good agreement with the available experimental data, remarkably improving over pure electronic computations, but also with respect to vibronic spectra obtained neglecting inter-state couplings. Our QD simulations indicate an effective population transfer from the lowest energy bright excited states to the close-lying dark excited states for uracil, thymine and adenine. Dynamics from higher-energy states show an ultrafast depopulation toward the more stable ones. The proposed protocol is sufficiently general and automatic to promise to become useful for widespread applications.

## 1. Introduction

The accurate description of the photoactivated dynamics of heterocyclic molecules is a very challenging task. The presence of the heteroatoms’ Lone Pairs (LPs) leads to the appearance of several low-lying n$\pi^{*}$ transitions, which, together with Rydberg states, usually make the Franck–Condon (FC) region very ’crowded’, i.e., with many possible closely-lying excited states that can interact with the spectroscopic bright $\pi \pi^{*}$ transitions. A fully resolved description of the excited Wave Packet (WP) dynamics, characterizing all the population transfer occurring after excitation, is therefore not straightforward. DNA and RNA nucleobases are prototypical heterocylic compounds and therefore it is not surprising that many details of their photophysics are not yet understood and subject to very lively debate [1,2,3,4,5]. The presence of inter-state vibronic interactions can be guessed already from the absorption spectra of the molecules, which are often broad and not well resolved even in the gas phase. For instance, in ref. [6] we showed that the ultrafast internal conversion (IC) from the bright $\pi \pi^{*}$ states to dark n$\pi^{*}$ states following photoexcitation of cytosine in gas-phase is responsible for a loss of vibronic resolution and broadening of the spectrum, as observed in the experiment. Recent methodological advances have made available a number of effective time-independent (TI) [7,8,9] and time-dependent (TD) [10,11,12,13,14,15] approaches for the calculation of the shape of electronic spectra, fully accounting for vibronic effects in rigid systems, meaning those well described by harmonic potential energy surfaces (PESs), if nonadiabatic interactions are negligible. Linear vibronic coupling (LVC) and quadratic vibronic coupling (QVC) models represent the natural generalization of the Hamiltonian for rigid systems which, on the contrary, undergo remarkable inter-state couplings [16,17]. They have attracted a large interest recently [18,19,20,21,22,23]. TI approaches for the calculation of the spectra are based on the knowledge of all the relevant vibronic eigenstates. Therefore their application to nonadiabatic systems is impractical, if not impossible, if more than a few vibrations need to be considered. On the contrary, TD approaches are still effective also in these cases, since they are eigenstate-free and can compute the necessary time-correlation functions by numerically propagating vibronic WPs on coupled PESs. In this respect, over the last few decades multiconfigurational TD Hartree (MCTDH) [24,25], and its multilayer extension (ML-MCTDH) [26,27] have emerged as methods capable of simulating the coupled quantum dynamics (QD) of tens of vibrational modes and multiple electronic states. Density functional theory (DFT) and its TD extension (TD-DFT) for excited states have been probably the most popular electronic structure methods for the computation of vibronic spectra, since energies, gradients, and Hessians are available, allowing a good compromise between accuracy and computational time [28]. We have recently proposed [29] a general scheme for the parameterisation of LVC Hamiltonians from TD-DFT calculations, based on a maximum overlap criterion, that is effective enough to generate automatically the parameters for systems with dozens of normal modes and 10–20 coupled electronic states [21]. Besides their interest for spectroscopy, LVC Hamiltonians can reliably describe the photodynamics in semi-rigid systems, such as in the DNA/RNA bases, on the ∼100 fs time scale, i.e., before large amplitude motions are likely to take place [6,29,30,31]. Therefore, they offer a powerful tool to investigate the initial steps of the intricate dynamics triggered in heterocyclic compounds by light absorption.

In this study, we utilise LVC models parameterised with TD-DFT in combination with ML-MCTDH wavepacket propagations in order to extend our previous study on cytosine (Cyt) [6] to analyze the importance of the vibronic interactions in determining the absorption spectral shapes of the other four nucleobases, i.e., the pyrimidines uracil (Ura) and thymine (Thy), and the purines adenine (Ade) and guanine (Gua), including both 7H and the 9H tautomers of Gua. Whilst the focus of the present study is on the spectroscopy, we shall also briefly analyse the nonadiabatic dynamics for each of the bases, focusing in particular on its dependence on the excitation energy. For the first time in fact, we shall systematically and individually simulate the dynamics starting from the 2/3 lowest energy bright diabatic excited states, which enable to monitor all population encompassed in the two lowest energy experimental absorption bands and check for the effect of the excitation wavelength on the dynamics. As mentioned above and discussed further below, an LVC Hamiltonian is expected to deliver reliable results on the 100 fs timescale, while at longer times the lack of possible additional decay mechanisms, and the inadequate description of possible large amplitude out-of-plane motions make the predictions progressively less robust. However, this time-window not only rules the shape of the absorption spectra, but is also critical for the entire dynamics, since it can determine the inertia of the WP. Being focused mainly on the analysis of the spectra, triplet states are not included in our study, since their participation to the dynamics becomes relevant after a few hundreds of fs. [5,32,33,34]. We shall parameterise the LVC model by using two different functionals (CAM-B3LYP and PBE0), since, as shown by our very recent studies on cytosine derivatives [29,31], they provide a different description of the relative stability of the lowest lying dark n$\pi^{*}$ excited states. The comparison of their predictions thus provides an important test of the ‘robustness’ of our results and, at the same time, interesting insights on the dependence of the dynamics on the inter-state vibronic couplings. Besides its methodological relevance, this study contributes an interesting opportunity for a quick re-assessment of the photoexcited behavior of nucleobases. This is an important step towards a full understanding of the molecular mechanism underlying the photodamage/photostability of nucleic acids upon UV irradiation, which is a relevant topic for biochemical and biomedical research.

## 2. Materials and Methods

### 2.1. Diabatisation and Linear Vibronic Coupling Model

The LVC Hamiltonian for a set of coupled electronic diabatic states, $|d\rangle =(|d_{1}\rangle ,|d_{2}\rangle ,$$\ldots ,|d_{n}\rangle )$, in dimensionless normal coordinates q (calculated on the ground state, *g*), with associated momenta p is given by

(1) $$\begin{array}{c} H=\sum_{i}\left(K+V_{ii}^{\mathrm{dia}}\left(q\right)\right)|d_{i}\rangle \langle d_{i}|+\sum_{i,j>i}V_{ij}^{\mathrm{dia}}\left(q\right)\left(|d_{i}\rangle \langle d_{j}|+|d_{j}\rangle \langle d_{i}|\right) \end{array}$$

where the kinetic (*K*) and potential (*V*) energy terms are defined as follows:

(2) $$\begin{array}{ccc} K & = & \frac{1}{2}p^{T}\Omega p \end{array}$$

(3) $$\begin{array}{ccc} V_{ii}^{\mathrm{dia}}\left(q\right) & = & E_{i}^{0}+\lambda_{ii}^{T}q+\frac{1}{2}q^{T}\Omega q, \end{array}$$

(4) $$\begin{array}{ccc} V_{ij}^{\mathrm{dia}}\left(q\right) & = & \lambda_{ij}^{T}q. \end{array}$$

Ω is the diagonal matrix of the vibrational frequencies of the ground state *g*, $E_{i}^{0}$ is the *i*th excited-state energy at the *g* equilibrium geometry, $\lambda_{\mathrm{ii}}$ is the energy gradient of state *i* (describing a shift of the equilibrium position) and $\lambda_{\mathrm{ij}}$ is the gradient of the inter-state coupling $V_{ij}^{\mathrm{dia}}\left(q\right)$. The PES of diabatic state *i*, $V_{ii}^{\mathrm{dia}}\left(q\right)$, is a quadratic function of q with the same normal modes and frequencies of *g* and the inter-state couplings $V_{ij}^{\mathrm{dia}}\left(q\right)$ have a linear dependence with q.

To calculate the parameters $\lambda_{\mathrm{ij}}$ we used a diabatization procedure based on maximum overlap of the set of diabatic reference states (defined as coincident with the adiabatic electronic states |a〉 at *g* equilibrium geometry) with the diabatic states obtained for displaced structures along each normal coordinate α by a small quantity $\pm \Delta_{\alpha }$

(5) $$|d(\Delta_{\alpha })\rangle =D(\Delta_{\alpha })|a(\Delta_{\alpha })\rangle .$$

For the displaced structures, the diabatic states are defined as the linear combination of adiabatic states that resemble as much as possible to the reference states. The transformation matrix $D(\Delta_{\alpha })$ can then be applied to the diagonal matrix of adiabatic energies

(6) $$V^{\mathrm{ad}}\left(\Delta_{\alpha }\right)=\mathrm{diag}\left(E_{1}^{\mathrm{ad}}\left(\Delta_{\alpha }\right),E_{2}^{\mathrm{ad}}\left(\Delta_{\alpha }\right),\ldots ,E_{n}^{\mathrm{ad}}\left(\Delta_{\alpha }\right)\right),$$

such that the matrix of diabatic potentials is

(7) $$V^{\mathrm{dia}}\left(\Delta_{\alpha }\right)=D^{⊺}\left(\Delta_{\alpha }\right)V^{\mathrm{ad}}\left(\Delta_{\alpha }\right)D\left(\Delta_{\alpha }\right).$$

The parameters $\lambda_{\mathrm{ij}}$ may then be calculated by numerical differentiation

(8) $$\lambda_{ij,\alpha }=\frac{\partial V_{ij}^{\mathrm{dia}}\left(q\right)}{\partial q_{\alpha }}≃\frac{V_{ij}^{\mathrm{dia}}\left(\Delta_{\alpha }\right)-V_{ij}^{\mathrm{dia}}\left(-\Delta_{\alpha }\right)}{2\Delta_{\alpha }}.$$

In this scheme, the $E_{i}^{0}$ in Equation (3) are therefore equal to the adiabatic excitation energies at *g* equilibrium geometry. For a full derivation of the method we refer the reader to our previous works [6,29,31].

### 2.2. Absorption Spectra

The absorption spectra ϵ(ω) at zero Kelvin, can be expressed in a TD framework as:

(9) $$\begin{array}{ccc} \epsilon (\omega ) & = & \frac{2\pi \omega N_{A}}{3000\times \ln10\times \hbar c_{0}\left(4\pi \epsilon_{0}\right)}\sum_{ji}\int_{-\infty }^{\infty }dte^{i\omega t-\Gamma t^{2}}\langle 0;d_{j}|\mu_{gj}e^{-i\hat{H}t/\hbar }\mu_{ig}|d_{i};0\rangle \\ & = & \sum_{i}\epsilon_{ii}\left(\omega \right)+\sum_{i,j\neq i}\epsilon_{ij}\left(\omega \right)=\epsilon^{\mathrm{auto}}\left(\omega \right)+\epsilon^{\mathrm{cross}}\left(\omega \right) \end{array}$$

where $N_{A}$ is Avogadro’s number, $c_{0}$ is the speed of light in vacuo, $\epsilon_{0}$ is the vacuum permittivity and we introduced a quadratic damping ruled by a parameter Γ, corresponding to a Gaussian broadening in the frequency domain. The electric transition dipole moment between state *g* and $d_{j}$ is given by $\mu_{gj}=\langle g|\mu |d_{j}\rangle$ and is considered independent of the nuclear coordinates (Condon approximation); the ground-vibrational state of the ground electronic state is represented by 0 and its energy is set to zero. The auto ($\epsilon^{\mathrm{auto}}$) and cross ($\epsilon^{\mathrm{cross}}$) correlation functions are obtained by numerical propagation in time under the effect of the LVC Hamiltonian of the doorway states $|d_{j};0\rangle$ obtained by a vertical excitation of the vibrational state 0 to the bright diabatic states (i.e., for which $\mu_{ig}$ is non vanishing). Then, the vibronic absorption spectrum is obtained by Fourier transform of the sum of the correlations functions weighted by the scalar products of the transition dipoles (as reported in Equation (9)). Cross-correlations functions usually have a very small effect and have been neglected [21,30].

## 3. Computational Details

Electronic structure calculations have been performed with DFT for the ground states, and TD-DFT for the excited states, using CAM-B3LYP [35] and PBE0 functionals [36,37], with 6-311+G(d,p) and 6-31G(d) basis sets in the Gaussian 16 program [38]. The former basis set is used in the main text, whilst results from the latter may be found in the Supporting Information (SI). TD-DFT computations were performed using tight SCF convergence, with a 10${}^{-6}$ a.u. threshold.

**Figure 1:**
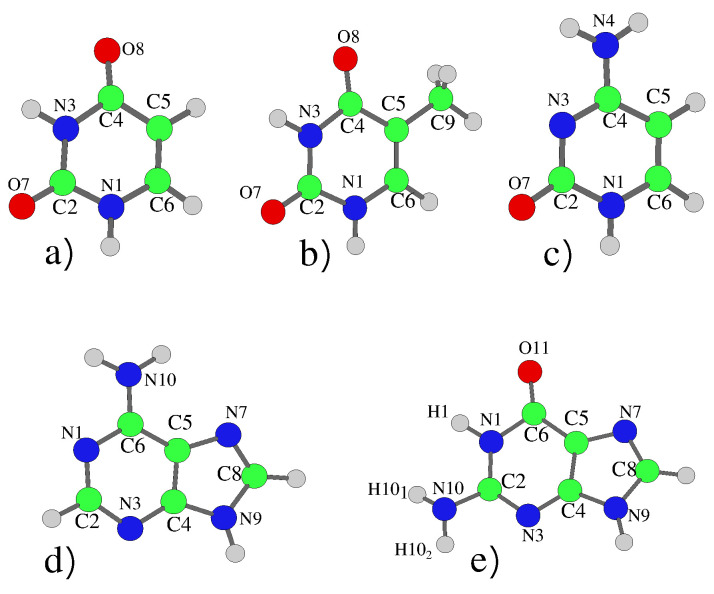
Schematic description (and atom labeling) of the five nucleobases: (**a**) Uracil, (**b**) Thymine, (**c**) Cytosine, (**d**) Adenine and (**e**) 9H-Guanine. In the 7H tautomer the hydrogen atom is bonded to the N7 Nitrogen atom rather than N9.

The nucleobases are shown in Figure 1, with geometries optimised adopting $C_{s}$ symmetry, which permitted electronic decoupling of the $\pi \pi^{*}$ (A’) and n$\pi^{*}$ (A”) states. For Cyt, Ade and Gua this resulted in small imaginary frequencies corresponding to the pyramidalization of the nitrogen of the amino group. For Cyt, simply taking the absolute value of these frequencies has previously been shown to not affect the resulting dynamics [6,29]. For the purine bases, we tested relaxing the symmetry constraint and thus obtaining a true-minimum geometry for 9H-Gua. The resulting dynamics and spectra were not significantly affected, the main difference was the energy of the lowest Rydberg state being lowered by ∼0.1 eV, due to the virtual orbital being located around the amino group, leading to some mixing with the lowest bright state (see SI Sections S1.5 and S2.5).

The vibrational frequencies obtained in the S${}_{0}$ state are utilised for each of the excited states in the LVC models, which includes both the in-plane (A’) and out of plane (A”) modes, thus providing a full dimensional picture for each of the nucleobases. Concerning the pyrimidine bases, the lowest three bright $\pi \pi^{*}$ states are included for Ura and Thy in the LVC models, and lowest two bright $\pi \pi^{*}$ states for Cyt, whilst for the purine bases the lowest two bright $\pi \pi^{*}$ states, commonly referred to as the L${}_{a}$ and L${}_{b}$ are included. For all bases 3 dark n$\pi^{*}$ states are included, except for 7H-Gua, as mixing of the lone pairs on O and N atoms results in the third n$\pi^{*}$ state lying higher in energy than the third $\pi \pi^{*}$ state, which we do not consider in this work. Rydberg (πRy${}_{\sigma }$) states are also included for the LVC models parameterised with the 6-311+G(d,p) basis sets. The number of πRy${}_{\sigma }$ states depends upon the nucleobase and functional. Tables reporting the nature of the electronic states, together with their energies, oscillator strengths, predominant orbital transitions and natural transition orbitals (NTOs) are given in the SI, Section S1 (Supplementary Materials). The diabatisation was performed with an in-house code that is interfaced with Gaussian 16 and freely available upon request.

Quantum dynamics calculations were performed with the ML-MCTDH method [26,27,39,40], adopting the implementation within the Quantics package [41]. We used a variable mean field (VMF) with a Runge–Kutta integrator order of 5 and accuracy 10${}^{-7}$, as in the provided examples for S${}_{2}$/S${}_{1}$ dynamics of pyrazine with 24 normal modes [40,42]. For the primitive basis set we adopted Hermite DVR functions, as appropriate for harmonic potentials. We checked convergence by monitoring the populations at the beginning and end of the grid using the rdgpop tool provided in Quantics, and ensuring that they did not exceed 10${}^{-9}$. For the ML tree, we chose the number of single particle functions (SPFs) for each node based on the magnitude of the on-diagonal vibronic coupling $\lambda_{ii}$ at the FC point, with modes with larger couplings assigned larger numbers of SPFs, as we have done in previous studies [6,29,30,31]. Mode combination was also utilised for modes with similar character. The eigenvalues of the density matrices of each node in the ML tree, also known as the natural weights, were monitored, ensuring that the upper natural weight was always less than 1% to obtain a reasonable quality calculation, as indicated in the Quantics manual.

For the absorption spectra, the dynamics calculations were initiated on the bright (all A’) and the quasi-dark (typically A” states), to establish whether any small contributions arose from the latter. As we have shown previously that the cross-correlation function provides only a negligible contribution to the spectra [6], the absorption spectra were calculated from the auto-correlation functions produced by the dynamics calculations, as discussed in Section 2.2, with phenomenological Gaussian broadening of 0.04 eV.

In order to monitor the effect of inter-state couplings, spectra are also calculated by switching them off before running the dynamics calculations (i.e., $V_{ij}^{\mathrm{dia}}=0$). This is equivalent to the Vertical Gradient (VG) approach [43]. Finally, pure electronic spectra are also presented from TD-DFT excitation energies and oscillator strengths, by simply broadening each stick line with Gaussian lineshape with a half width at half maximum (HWHM) of 0.25 eV. A “pure electronic” spectrum is the simplest implementation of what is known as the classical FC principle [44]. More sophisticated approaches obtain the spectrum from the ensemble of transition energies and intensities at a representative number of nuclear structures (nuclear ensemble approach, NEA) [45]. In harmonic models, like the ones we are considering here, even the quantum Wigner distribution of nuclear structures is analytically known. The computation of the spectrum from a sampling of the Wigner distribution guarantees to recover exactly the first and second moment of the quantum vibronic spectrum (for transition dipoles independent of the coordinates) [44]. However vibronic structures cannot be described and higher moments (e.g., the asymmetry) are not reproduced. The application of NEA is very interesting in combination with an explicit repetition of the electronic calculations at all configurations. This strategy in fact allows to approximately describe the possible effects of anharmonicity [46,47,48], and it opens the route to the description of systems in explicit environments (with QM/MM methods). Therefore, many interesting progresses have been recently reported for NEA. Just to quote a few of them, it has been shown that quantum distributions can be obtained also for non-harmonic cases by path integral dynamics [47,49], and that convergence can be remarkably sped up with machine learning techniques [50]. Finally, mixed quantum classical nuclear-ensemble/vibronic approaches have been recently proposed to compute the spectra of flexible systems in explicit environments [46,48]. For the analysis we present here, where we invoke the harmonic approximation for all electronic states, the quantum vibronic results we report provide the "exact" spectra. In this context, the detailed comparison with the classical approximation based on Wigner distribution is outside the main scopes of the paper. In fact, differences in the expected results of classical and quantum computations of the spectra have been already analysed several times in the literature (see for instance ref. [51]). For the sake of completeness, we report such an analysis just for Ura in the SI, Section S2.1, while in the main text we only compare vibronic LVC calculations (all vibrational effects included) with the simple “pure-electronic” spectrum (no vibrational effect included). The computed spectra are displaced along the energy axis for best comparison to experimental results [52,53], with shifts indicated in the relevant figure captions.

Despite, as we mentioned above, the adoption of a LVC Hamiltonian restricts the full reliability of our predictions to the ∼100 fs time-scale, i.e., before large amplitude motions take place, in the following we plot the time evolution of the electronic populations up to 250 fs. In fact it is interesting to check if, within the assumptions of the model, the electronic populations are expected to exhibit remarkable features, like for instance quantum beats, at longer times. Results will show in most of the cases large quantum beats are not observed, indicating that, even in gas phase, the multimode dynamics is complicated enough to induce an effective dephasing. The ∼100 fs timescale is more than sufficient for a fully-converged calculation of the spectrum with the selected frequency resolution.

## 4. Results

### 4.1. Pyrimidines

#### 4.1.1. Uracil and Thymine

The lowest energy excited states in the FC region for Ura and Thy are quite similar both in character and energy (see Table 1, full Tables in SI, Sections S1.1 and S1.2). In agreement with many previous computational studies [1], S${}_{1}$ is a dark state with n$\pi^{*}$ character that can be described as arising from the excitation from the LP of the C4-O8 carbonyl group to the $\pi^{*}$ orbital. As a consequence we label it as n${}_{O}\pi^{*}1$. The lowest energy bright excited state ($\pi \pi^{*}1$) corresponds instead to the HOMO→LUMO $\pi \pi^{*}$ transition. Table 1 shows that the 5-methyl substituent in Thy leads simply to a weak red-shift of the bright excited states involving the HOMO, $\pi \pi^{*}1$ and $\pi \pi^{*}3$, with the consequent decrease of the energy gap $\pi \pi^{*}1$/$n_{O}\pi^{*}1$. Concerning the effect of basis set (see Tables S1 and S6 in the SI), the n$\pi^{*}$ states are predicted to be similar in energy (within ∼0.05 eV) by both 6-311+G(d,p) and 6-31G(d) basis sets, whilst the $\pi \pi^{*}$ states are ∼0.1–0.3 eV blue-shifted by the 6-31G(d) basis set.

**Figure 2:**
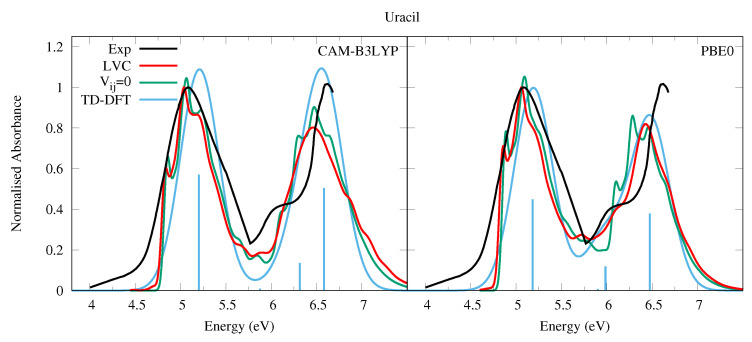
Absorption spectra of Uracil from the LVC model (red), without inter-state coupling (green), TD-DFT calculations with stick spectra (blue), and experimental data (black) [52]. Cusp in experimental spectrum at 5.75 eV due to gap in data from 5.5 eV to 5.75 eV as separate spectra obtained for lower energy and upper energy bands, recorded at different temperatures (501 K and 439 K, respectively). Low energy peak from experiment and LVC spectra normalised to 1, with other calculated spectra normalised with the same value as LVC spectra. CAM-B3LYP spectra red-shifted by 0.3 eV, PBE0 by 0.15 eV. LVC spectra broadened with Gaussian HWHM 0.04 eV, TD-DFT with HWHM 0.25 eV.

The experimental absorption spectrum of Ura in the vapor phase (at 501 K for the lower energy band, 439 K for the higher energy band, see the caption of Figure 2) exhibits a broad band peaking at ∼5.1 eV (244 nm, band I) with a red-wing tail. At higher energy a band with a maximum at 6.6 eV is present, though the spectrum is not very well resolved in this region. In water there are two bands with similar intensities, peaking at ∼4.8 eV and ∼6.1 eV [54]. The computed spectra are in excellent agreement with the experimental ones, when considering that the shape of the second energy band is likely affected by higher energy transitions not included in our treatment [55]. As shown in Figure 2 we predict two bands of similar intensities, weakly blue-shifted with respect to the experiments, i.e., ∼0.3 eV and ∼0.15 eV according to CAM-B3LYP and PBE0, respectively. For what concerns the comparison with the spectra measured in water, it has been shown that inclusion of solvent effect leads to a small red-shift of the $\pi \pi^{*}1$ transition energy [1,56,57,58,59,60].

The comparisons with the spectra simulated by convolution of the electronic stick lines with a phenomenological Gaussian (blue curve in Figure 2) highlights the importance of a proper inclusion of vibrational effects, showing that the vibrationally resolved spectra are in a significantly better quantitative agreement with the experimental shape. Focusing on the lowest-energy band: first, a spectral width comparable with experiment is obtained with a minimal phenomenological broadening (HWHM = 0.04 eV) to be compared with the large HWHM = 0.25 eV adopted in pure electronic spectrum. Second, despite the large phenomenological broadening, the shape of the pure-electronic spectrum clearly does not fit the experimental one, while the vibronic spectrum better reproduces the asymmetric experimental shape. Additionally, the computed maximum of band I is 0.1∼0.15 eV closer to the experimental maximum. This vibrational red-shift has also been observed recently in a TI approach to computing the absorption spectra of Uracil [60] and the DNA bases [59]. It is important to remember that our treatment neglects Duschinsky mixing and the effect of temperature, both of which are expected to further broaden the spectra and red-shift the maxima, better improving the agreement with the experimental spectra. Actually the effect of Duschinsky mixing on the $\pi \pi^{*}1$ state only (neglecting inter-state couplings) was illustrated in Ref. [43] comparing spectra computed with the VG and Vertical Hessian (VH) vibronic models. Further broadening might be due to the intrinsic tendency of $\pi \pi^{*}1$ to distort out of planarity (in C${}_{s}$ it shows 1–2 imaginary frequencies [43], which would require an anharmonic treatment). Finally, vibronic couplings allow to partially recover (although largely underestimate) the significant experimental absorption in the 5.8–6 eV region, which is absent in the purely electronic spectrum.

The effect of the inter-state coupling on the spectra can be appreciated by looking at the green lines in Figure 2, computed by switching off the inter-state coupling. The green spectrum shows a slightly more pronounced vibronic resolution and, in particular, the red-wing tail falls more rapidly. This contribution is related to the coupling between $\pi \pi^{*}1$ and n${}_{O}\pi^{*}$1. On the other hand, the experimental red-wing tail is much larger than in the simulated spectra. This discrepancy indicates it is likely that the coupling between the bright and the n${}_{O}\pi^{*}$1 state is underestimated at our level of theory. It is also likely that Duschinsky and thermal effects would enhance the coupling. The latter is suggested by the fact that at 501 K the two lowest frequency A” vibrational modes that couple the $\pi \pi^{*}$and n$\pi^{*}$states will be thermally excited.

**Figure 3:**
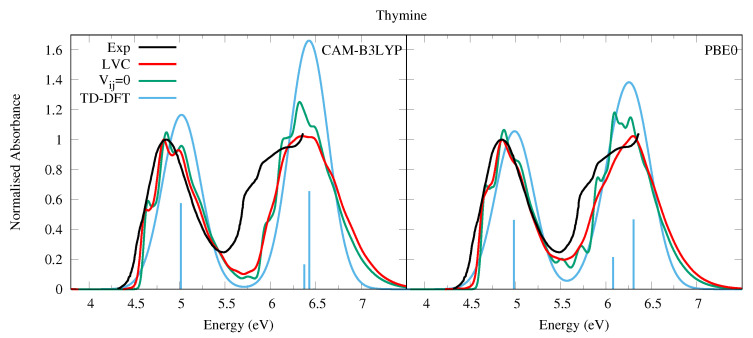
Absorption spectra of Thymine from the LVC model (red), without inter-state coupling (green), TD-DFT calculations with stick spectra (blue), and experimental data of 1-methylthymine (black) [52]. Low energy peak from experiment and LVC spectra normalised to 1, with other calculated spectra normalised with the same value as LVC spectra. CAM-B3LYP spectra red-shifted by 0.3 eV, PBE0 by 0.15 eV. LVC spectra broadened with Gaussian HWHM 0.04 eV, TD-DFT with HWHM 0.25 eV.

We did not succeed in finding in the literature a sufficiently well-resolved absorption spectrum in the gas phase (and covering the entire 200–300 nm region) of Thy, therefore instead in Figure 3 we present that of 1-methylthymine taken at a temperature of 369 K [52]. This is very similar to that of Ura, but for a weak red-shift, and a slightly closer spacing of the high and low energy bands. In methylcyclohexane two bands of similar intensity peaking at ∼4.7 and ∼6.0 eV are observed [54]. The simulated spectra for Thy are very similar to the experimental one and the conclusions of our analysis of Ura spectra are fully confirmed. The LVC spectra, which are in good quantitative agreement with experiments, are red-shifted and significantly broader than that obtained by convolution of the stick transitions. Moreover, the vibronic contributions explain the weak shoulder observed in the experiment at ∼4.7 eV assigning it to the 0-0 vibronic transition.

PBE0 better reproduces the shoulder at ∼5.8 eV, likely due to $\pi \pi^{*}2$, which is instead too close to $\pi \pi^{*}3$ according to CAM-B3LYP, leading also to the overestimation of the intensity of the band peaking at ∼6.5 eV. As a consequence, the purely electronic spectrum also remarkably overestimates the maximum intensity of the second band with respect to the first one. The residual discrepancy of the PBE0 spectrum with experiment suggests that the computed energy for $\pi \pi^{*}2$ is still too high and its intensity too weak. Furthermore, for Thy, like with Ura, the absence of thermal and Duschinsky effects in the LVC spectrum result in an underestimation of the broadness of the spectral bands and the intensity of the red-wing tail with respect to the experiments. However, in this case such a tail is much less extended, probably as an effect of the lower temperature the 1-methylthymine spectrum was taken, and the reduced gap between the $\pi \pi^{*}$1 and n${}_{O}\pi^{*}$1 states in Thy due to the slight red-shift of $\pi \pi^{*}$1 (Table 1).

**Figure 4:**
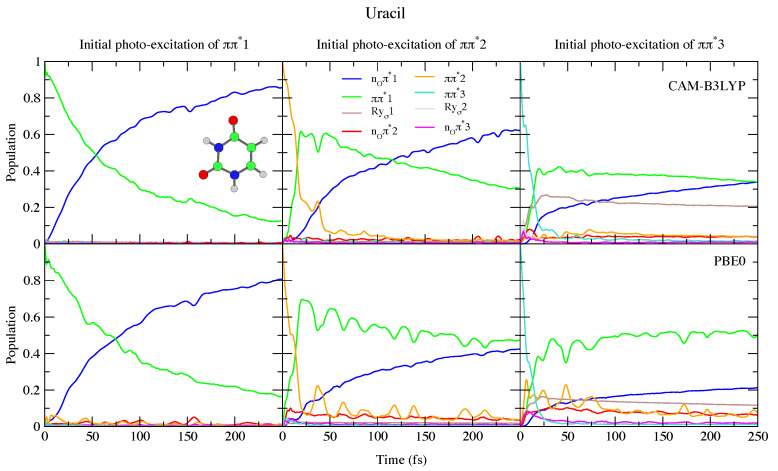
Nonadiabatic dynamics of electronic populations of Uracil in the gas phase, as predicted by an LVC Hamiltonian parameterised with calculations at the FC point using CAM-B3LYP (**top**) and PBE0 (**bottom**) functionals with a 6-311+G(d,p) basis set.

**Figure 5:**
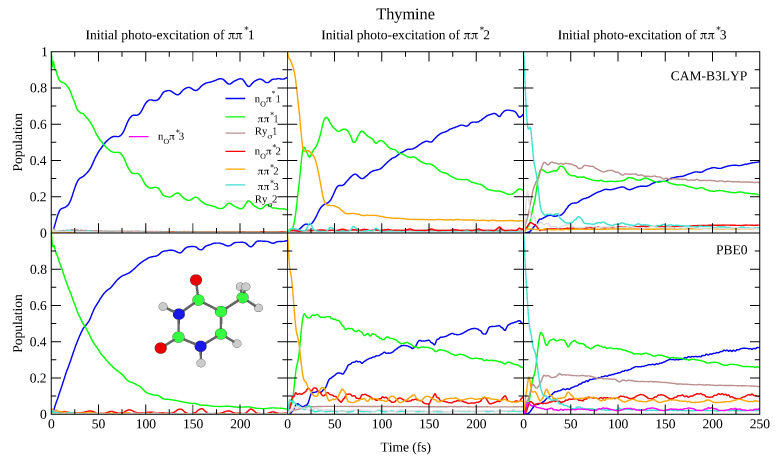
Nonadiabatic dynamics of electronic populations of Thymine in the gas phase, as predicted by an LVC Hamiltonian parameterised with calculations at the FC point using CAM-B3LYP (**top**) and PBE0 (**bottom**) functionals with 6-311+G(d,p) basis set.

This reduced gap, as well as the slight red-shift of $\pi \pi^{*}3$ in Thy with respect to Ura has a small effect on the dynamics shown in Figure 4 and Figure 5. Independently of the computational model (see Figures S15 and S18 in the SI for results with 6-31G(d) basis set) for both bases we observe a very effective $\pi \pi^{*}1\to$n${}_{O}\pi^{*}1$ population transfer. Within ∼50–70 fs, 50% of the population has decayed to n${}_{O}\pi^{*}1$, and only for Ura/PBE0 is this transfer slightly slower. This result is in excellent agreement with the 60 ± 30 fs decay time measured for the $\pi \pi^{*}1\to$n${}_{O}\pi^{*}1$ process of Thy in gas phase by Wolf et al. [61]. In fact, an approximate mono-exponential fit of the computed $\pi \pi^{*}$ population for Thy gives a time constant τ∼66 and ∼47 fs, according to CAM-B3LYP and PBE0 (and a decay completed by 90% and 98%, respectively, see Figure S19 in the SI). More in general, keeping in mind that our simulations do not consider the $\pi \pi^{*}1\to$S${}_{0}$ non-radiative deactivation channel, which is instead expected quite effective and fast [1], our predictions are fully consistent with the studies in the literature (see refs. [33,34,60,62,63,64,65,66,67,68,69,70,71,72,73,74,75] for a more detailed discussion). Decay constants for the higher energy bright states are predicted to be even faster, being ∼24 fs (CAM-B3LYP) and ∼11 fs (PBE0) for $\pi \pi^{*}2$, and ∼10 fs for $\pi \pi^{*}3$ (both functionals).

To the best of our knowledge the dynamics following individual excitation to $\pi \pi^{*}2$ and $\pi \pi^{*}3$ diabatic states, i.e., covering all the photoexcited population of the two lowest energy absorption bands, has never been investigated by calculations. Our simulations indicate that in both cases ∼50% of the population is transferred to $\pi \pi^{*}1$ within 25 fs. Then the systems proceed as described above after excitation to $\pi \pi^{*}1$, with a large population transfer to n${}_{O}\pi^{*}1$. However, in these cases, after 250 fs $\pi \pi^{*}1$ is still significantly populated, especially according to PBE0 and after excitation to $\pi \pi^{*}3$, with the population ∼40% for Ura and ∼25% for Thy in this case. Furthermore, for initial excitation to $\pi \pi^{*}$3, the lowest lying Rydberg state and, for PBE0 the n${}_{O}\pi^{*}$2 state, are somewhat populated after 250 fs. They are not predicted to play as large a role in the dynamics initiated on $\pi \pi^{*}$2, and the higher lying Rydberg and n${}_{O}\pi^{*}$3 states are not predicted to play a significant role in any of the calculations.

#### 4.1.2. Cytosine

**Figure 6:**
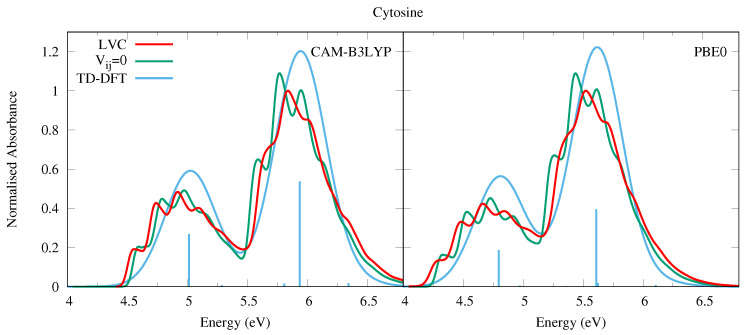
Absorption spectra of Cytosine from the LVC model (red), without inter-state coupling (green), and TD-DFT calculations with stick spectra (blue). Most intense peaks from LVC spectra normalised to 1, with other calculated spectra normalised with the same value. All calculated spectra unshifted. LVC spectra broadened with Gaussian HWHM 0.04 eV, TD-DFT with HWHM 0.25 eV.

In 2018, some of us have used the same methodology adopted here to compute the absorption spectrum of Cyt, considering all populated tautomers [6], and obtaining a nice agreement with the experimental spectra. More recently, we have investigated in detail the QD of the biologically relevant keto-amino tautomer of Cyt and of its methylated derivatives [29,31]. Nonetheless, for the sake of completeness, in Figure 6 we report the spectra computed for the keto-amino tautomer by using the same computational approach as for the other bases. Since three tautomers are present in the gas phase, and the the keto-amino is not the most populated one, the computed spectrum cannot be directly compared with the experiments (see our study in Ref. [6] and the works in refs. [76,77,78] for further details). Nonetheless, it is apparent that vibronic effects strongly impact the spectral shape, fully confirming the trends highlighted for Ura and Thy. Interestingly, notwithstanding the fact that the net population transfer to the dark states is smaller than for Ura and Thy, the excited state dynamics of Cyt is particularly rich, since several n$\pi^{*}$ transitions are close in energy to $\pi \pi^{*}1$ and $\pi \pi^{*}2$ (see Figure S21 in the SI). This feature has also been observed in previous dynamics studies [78,79,80,81]. Furthermore, the character of the n$\pi^{*}$ states is different with different functionals, although similar to Ura and Thy the effect of basis set is modest, see Table 1, Section S1.3 in the SI, and Ref. [29] for more details. Despite this, the spectra obtained via the CAM-B3LYP and PBE0 parameterisation are remarkably similar, and the inclusion of diabatic coupling has a clear effect on the spectral shapes, especially for the higher energy band.

### 4.2. Purines

#### 4.2.1. Adenine

The lowest three excited states for Ade predicted by CAM-B3LYP and PBE0 are consistent with previous studies [1], with an n$\pi^{*}$ state, and two $\pi \pi^{*}$ states (commonly referred to as L${}_{a}$ and L${}_{b}$ following Platt’s nomenclature) which are all close in energy, as shown in Table 2. The n$\pi^{*}$ state corresponds to a transition from the N1 and N3 lone pairs to the LUMO $\pi^{*}$ orbital (see Section S1.4 in the SI), and hence we label it n${}_{N}\pi^{*}$1. Furthermore, similar to previous studies with TD-DFT, the states follow the order *E*(n$\pi^{*}$) < *E*(L${}_{a}$) < *E*(L${}_{b}$) for both functionals (and basis sets, see Table S16 in the SI), and L${}_{a}$ has a large oscillator strength, whilst that of L${}_{b}$ is small.

**Figure 7:**
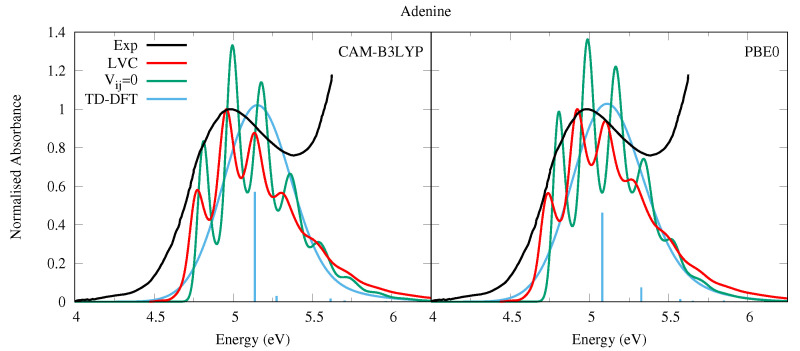
Absorption spectra of Adenine from the LVC model (red), without inter-state coupling (green), TD-DFT calculations with stick spectra (blue), and experimental data (black) [52]. Main peak from experiment and LVC spectra normalised to 1, with other calculated spectra normalised with the same value as LVC spectra. CAM-B3LYP spectra red-shifted by 0.25 eV, PBE0 by 0.08 eV. LVC spectra broadened with Gaussian HWHM 0.04 eV, TD-DFT with HWHM 0.25 eV.

The absorption spectra obtained by the LVC Hamiltonian are similar for both CAM-B3LYP and PBE0 parameterised models, shown in Figure 7. They are dominated by the absorption of the L${}_{a}$ state (see also Figure S24 in the SI where the individual contributions of each state are shown), and show an appreciable amount of vibronic resolution. Contrastingly, the experimental absorption spectrum of Ade in the vapor phase (at 500 K) exhibits a structureless band peaking at ∼5 eV, with a long red-wing tail [52].

The vibronic resolution in the LVC spectra is partially damped with respect to the spectra where inter-state coupling is switched off, illustrating the effect of coupling with the n${}_{N}\pi^{*}$1 state. Compared to the phenomenologically broadened TD-DFT stick spectra, the LVC spectra are significantly red-shifted, reducing the error with respect to the experimental spectrum. This finding confirms the trend already observed for the pyrimidine bases. Like for Ura, the lack of the long red-wing tail observed in the experimental spectrum suggests an underestimation of the L${}_{a}$/n${}_{N}\pi^{*}$1 coupling, whose effect is probably enhanced by the thermal excitation of the A” out-of-plane distortions at the high temperature at which the spectrum was measured. On the other hand, it is also possible that L${}_{b}$, whose relative stability with respect to L${}_{a}$ is still matter of debate [1,82], can contribute to this red-wing. We believe these explanations to be more likely than the presence of the 7H tautomer of Adenine, as we calculated the ground state (electronic plus zero point vibrational) energy of the 7H tautomer to be 36 kJ mol${}^{-1}$ greater than the 9H tautomer with CAM-B3LYP, and 35 kJ mol${}^{-1}$ greater with PBE0.

**Figure 8:**
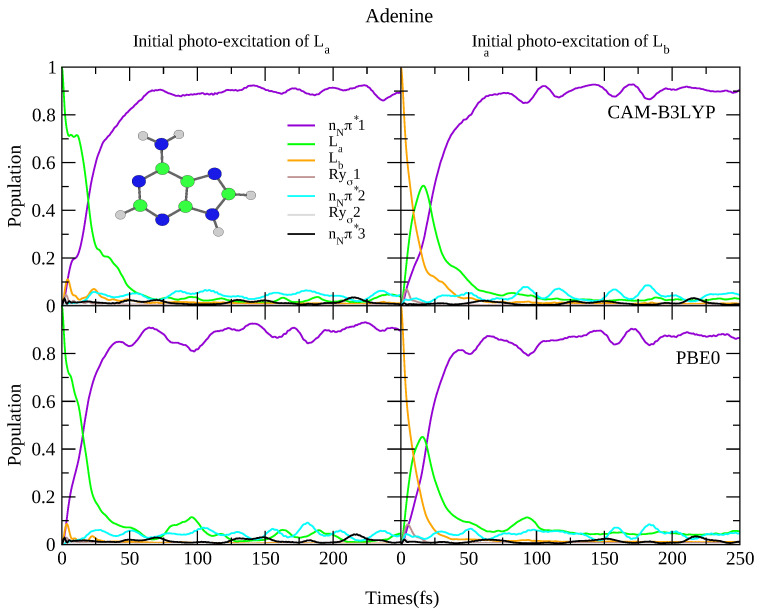
Nonadiabatic dynamics of electronic populations of Adenine in the gas phase, as predicted by an LVC Hamiltonian parameterised with calculations at C${}_{s}$ minimum using CAM-B3LYP (**top**) and PBE0 (**bottom**) functionals with 6-311+G(d,p) basis set.

For what concerns the dynamics, as shown in Figure 8 both functionals predict rapid transfer from the L${}_{a}$ state to n${}_{N}\pi^{*}$1, with ∼90% of the population transferred after 50 fs, and limited involvement of the other states. When initially excited to the L${}_{b}$ state, there is rapid transfer to the L${}_{a}$ state (almost all the population has transferred from L${}_{b}$ after 25 fs) concurrently with rapid transfer to the n${}_{N}\pi^{*}$1 state. This is consistent with previous dynamics studies observing rapid transfer to the n$\pi^{*}$ state [83,84,85,86], and experimental studies showing <100 fs decay constants [64,65]. It is also consistent with the smaller 6-31G(d) basis set, with the predicted dynamics virtually identical, shown in Figure S23 in the SI. Of course the fast L${}_{b}$→L${}_{a}$ transfer also arises from the fact that according to TD-DFT L${}_{a}$ is more stable than L${}_{b}$, differently from what predicted post-Hartree Fock methods [1], like CASPT2 [87], and coupled cluster [88]. Finally, once more we re-iterate that we do not consider excited state deactivation to the ground state, which as been analysed in detail in Ref. [89].

#### 4.2.2. Guanine

Inspection of Table 2 highlights that 9H-Gua exhibits a number of remarkable differences with respect to 9H-Ade. First, the energies of the L${}_{a}$, L${}_{b}$ and n$\pi^{*}$ states are more separated. Second, the n$\pi^{*}$state involves an excitation from the oxygen lone pair, rather than a nitrogen one, and it is not the lowest excited state (S${}_{3}$ with CAM-B3LYP and S${}_{4}$ with PBE0). Third, the L${}_{b}$ state now has a larger oscillator strength, roughly twice that of the L${}_{a}$ state. Moreover, the presence of a low lying Rydberg state is also observed with a diffuse basis set, with CAM-B3LYP predicting it to be S${}_{2}$ at the FC point, whilst PBE0 predicts it to be S${}_{1}$ (the Rydberg states are not observed for the smaller 6-31G(d) basis set, see Table S18 in the SI). All of this is consistent with previous studies [1], indicating that a Rydberg transition is extremely close to L${}_{a}$, their relative stability changing depending on the adopted ab initio method. For example, EOM-CCSD/aug-cc-pVTZ predicts that the Rydberg state is 0.03 eV more stable than L${}_{a}$, but, after inclusion of triple corrections, L${}_{a}$ is more stable by 0.04 eV [90]. This prediction is extremely close to that provided by CAM-B3LYP, which, thanks to the presence of long-range corrections, is expected to treat Rydberg states more reliably than PBE0.

Another difference with respect to Ade is that the 7H tautomer is predicted to be of a similar stability to the 9H one, with the ground state energy being 0.14 kJ mol${}^{-1}$ lower for the 7H tautomer with CAM-B3LYP and 1.0 kJ mol${}^{-1}$ lower with PBE0. This is similar to previous B3LYP/def2-TZVPPD calculations that predicted the 7H tautomer to be more stable by 3 kJ mol${}^{-1}$ [91]. In 7H-Gua, relative to 9H-Gua, the L${}_{a}$ state is red-shifted by ∼0.3 eV, whilst the L${}_{b}$ state is blue-shifted by ∼0.2 eV for both CAM-B3LYP and PBE0. The Rydberg state is also slightly blue shifted (∼0.1 eV) for 7H-Gua, and is no longer the lowest excited state according to PBE0. The n${}_{O}\pi^{*}$ state becomes mixed with the lone pair on N9 (see NTOs in SI Sections S1.5 and S1.6) and red-shifted by 0.1 eV according to CAM-B3LYP and ∼0.2 eV according to PBE0.

**Figure 9:**
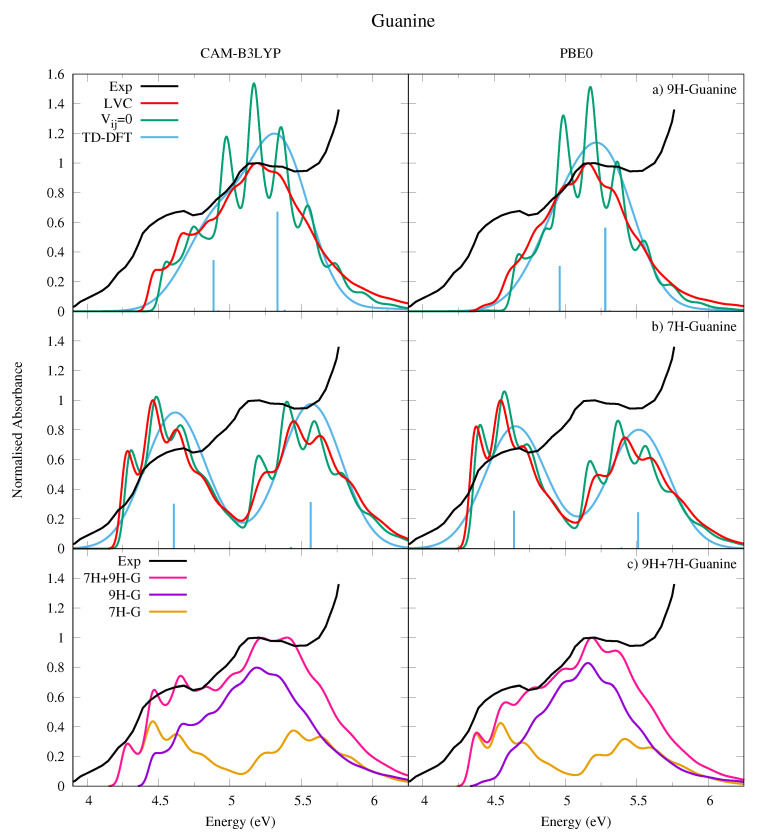
Absorption spectra of (**a**) 9H-Guanine and (**b**) 7H-Guanine from the LVC model (red), without inter-state coupling (green), TD-DFT calculations with stick spectra (blue), and experimental data (black) [53]. Most intense peak from experiment and LVC spectra normalised to 1, with other calculated spectra normalised with the same value as LVC spectra. Panel (**c**) shows the combination (pink) of 9H (purple) and 7H (orange) LVC spectra compared to experiment (black). Most intense peak from experiment and 7H-9H combination normalised to 1. Individual 7H and 9H spectra normalised with same value as 7H-9H combination. For all panels CAM-B3LYP spectra on the left red-shifted by 0.3 eV, PBE0 spectra on the right red-shifted by 0.08 eV. LVC spectra broadened with Gaussian HWHM 0.04 eV, TD-DFT with HWHM 0.25 eV.

We have computed the absorption spectra for both 9H-Gua in Figure 9a and 7H-Gua in Figure 9b, where they are compared to an experimental spectrum measured in a nitrogen matrix at 15 K [53]. We also report in Figure 9c a spectrum computed with the hypothesis that in the experimental conditions the population of 9H and 7H tautomers is exactly the same. Considering that the two tautomers are practically isoenergetic, it is not easy to estimate the tautomeric ratio in the nitrogen matrix, as it was prepared by directly subliming solid Gua into the matrix.

The experimental spectrum is characterized by a broad band with a first maximum at ∼4.6 eV and a second one, almost twice more intense, at ∼5.15 eV. The calculated spectrum for 9H-Gua in Figure 9a reproduces well the relative intensity of the high and low energy peaks, but not their separation. In contrast, the calculated spectrum for 7H-Gua in Figure 9b reproduces better the relative separation of the peaks, but not their intensities, as they are too similar. The spectrum computed by considering the contribution of both tautomers in Figure 9c however correctly reproduces both the relative intensity and the spacing of the two peaks, but for an uniform shift of ∼0.3 eV for CAM-B3LYP, and ∼0.1 eV for PBE0.

The lower energy band, attributed to L${}_{a}$, has a large red-wing tail, which our spectra indicates is predominantly due to 7H-Gua. 9H-Gua instead mostly contributes to the higher energy band, attributed to L${}_{b}$. It should be noted with reference to this assignment that it is also possible that the nitrogen matrix could affect the experimental spectrum. The shape of the high-energy region of the spectrum also clearly shows the effect of higher lying, very intense, excited states, not included in the present treatment.

Comparison with the spectra obtained without including inter-state coupling for each tautomer in Figure 9a,b shows the effect of the coupling between the L${}_{a}$ and L${}_{b}$ states, resulting in a large loss of vibronic resolution for the high energy peak, in particular for 9H-Gua (see also Figures S27 and S29 in the SI where the individual state contributions are shown). Relative to the LVC Ade spectra in Figure 7, the LVC 9H-Gua spectra are less structured, reflecting the greater contribution the L${}_{b}$ state has on the 9H-Gua spectrum. Moreover, in agreement with the trends already observed for the other bases the higher energy peaks undergo a greater loss of vibronic resolution due to strong inter-state couplings. Furthermore, mirroring trends observed for the other bases, the LVC spectra are red-shifted towards the experimental result relative to the TD-DFT phenomenologically broadened spectra, and the relative intensity of the higher and lower energy peaks are better reproduced.

**Figure 10:**
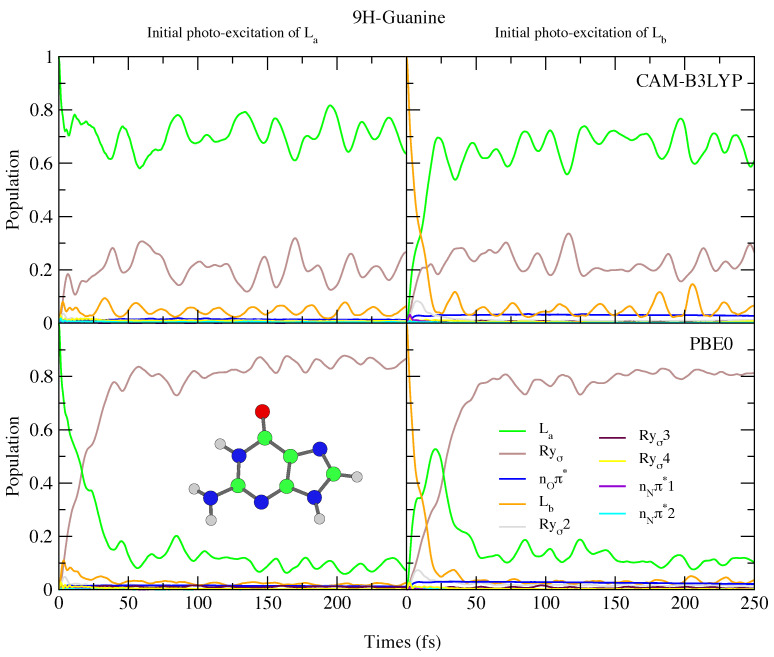
Nonadiabatic dynamics of electronic populations of 9H-Guanine in the gas phase, as predicted by an LVC Hamiltonian parameterised with calculations using CAM-B3LYP (**top**) and PBE0 (**bottom**) functionals with 6-311+G(d,p) basis set.

**Figure 11:**
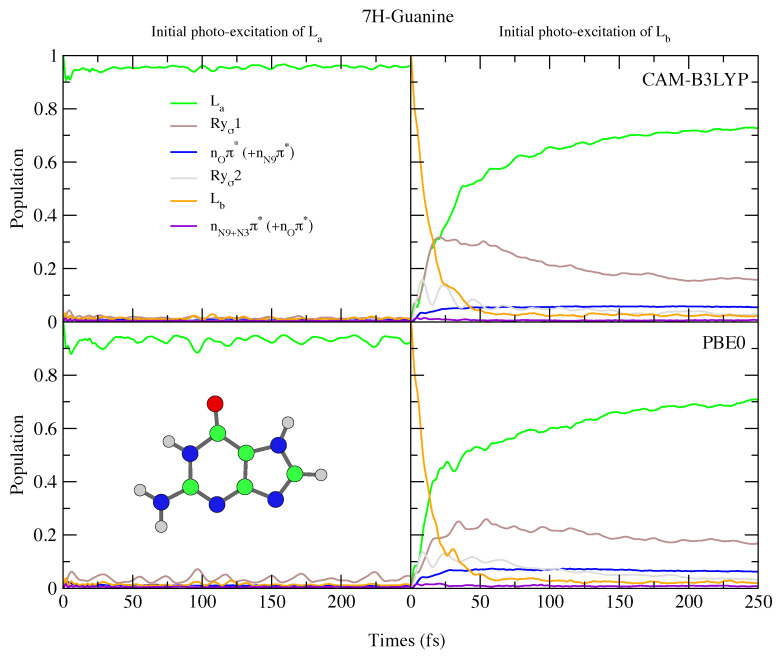
Nonadiabatic dynamics of electronic populations of 7H-Guanine in the gas phase, as predicted by an LVC Hamiltonian parameterised with calculations using CAM-B3LYP (**top**) and PBE0 (**bottom**) functionals with 6-311+G(d,p) basis set.

The photoexcited dynamics of 9H-Gua and 7H-Gua shown in Figure 10 and Figure 11 are quite different. We predict that for 9H-Gua there is a quite substantial involvement of the lowest Rydberg state, with ∼20% and ∼80% of the population transferred to it according to the simulations based on CAM-B3LYP and PBE0, respectively, both when initially exciting to L${}_{a}$ and L${}_{b}$. The parameterisation with the 6-31G(d) basis set (see Figure S25 in the SI) instead predicts the population to predominantly remain/be transferred to the L${}_{a}$ state, due to lack of Rydberg states in this model. The correlation of the Rydberg state with a N-H dissociative channel has been debated in the literature [92,93,94]. We do notice small elongations of the N1-H1 and N10-H10${}_{1}$ bonds in our calculations (see Figure 1 for the labels, and the SI Figure S26 and text surrounding for more details). On the other hand, also considering the limitations of our model, a detailed study of this issue falls outside the scope of the present paper.

Since in 7H-Gua L${}_{a}$ is red-shifted and the Rydberg state blue-shifted with respect to 9H-Gua, we predict that the Rydberg state is much less involved in the excited state dynamics. It is only ∼20% populated after initial excitation to L${}_{b}$, for both CAM-B3LYP and PBE0 models. Compared to Ade, there are also significant differences, with no involvement of the n$\pi^{*}$ states predicted for both 9H-Gua and 7H-Gua, in agreement with previous dynamics studies of 9H-Gua [95,96].

## 5. Concluding Remarks

In this work, we have calculated absorption vibronic spectra and ultrafast nonadiabatic dynamics triggered by light absorption of the 5 DNA and RNA nucleobases in the gas phase. For all bases we considered the first two or three lowest-energy bright states, and we accounted for all the couplings among them and with all the close lying dark states. In order to achieve this goal, we exploited the effectiveness of a recently proposed approach to parameterise LVC Hamiltonians with TD-DFT, and of the potentiality of QD nonadiabatic simulations conducted with the ML-MCTDH method.

The predicted absorption spectra are in good agreement with experiment, with a remarkable improvement over purely-electronic approaches, concerning the position, shape, width, as well as the relative intensity of the main bands. Our results clearly illustrate the effect of inter-state couplings, leading to fast IC, on the absorption spectra. They induce a broadening of the spectral profile and loss of vibronic resolution that is more significant for higher energy peaks. The residual differences with respect to the experimental shapes, mainly the too-high resolution of some of the computed peaks and the underestimation of the lowest-energy tail, are probably connected to the lack of thermal effects. In fact, calculations were performed at 0K but, for most of the nucleobases, experimental spectra in gas phase were only available at high temperatures. Apart from these small discrepancies, the nice comparison with the experimental spectra further supports the reliability of TD-PBE0 and TD-CAM-B3LYP for the study of nucleobases. TD-PBE0 spectra are, on the average, blue-shifted by 0.1∼0.15 eV with respect the experimental ones, whereas TD-CAM-B3LYP ones by ∼0.3 eV. The position of the computed spectra, which are expected to be further red-shifted by thermal and Duschinsky effects, are thus in almost quantitative agreement with the experimental ones.

To the best of our knowledge this is the first QD study reported for Gua, for both the 7H and 9H tautomers. In this respect, the comparison between the computed and the available experimental spectra strongly suggests that both tautomers are significantly populated in the gas phase. For Ura, Thy and Ade our computations predict an ultrafast (<100 fs) and almost complete decay of the first $\pi \pi^{*}$ state to dark n$\pi^{*}$ states, while such decay is only partial for the keto-amino tautomer of Cyt (and its extent is remarkably different according to PBE0 and CAM-B3LYP, see SI and a detailed discussion in ref [29]), and does not take place for the tautomers of Guanine. It is worthy to highlight that, in some cases, the predicted decays are so fast that, for a more reliable comparison, the characteristics (e.g., the duration) of the pump pulse adopted in the experiments should be accounted for in the computations too [97]. An interesting review of the theoretical dynamical studies on nucleobases and their comparison with available experiments updated to 2015 can be found in Ref. [5]. The data collected by the authors indicate that for all nucleobases, except Guanine, a sub 100 fs decay constant was measured at least in some experiments. Our QD results seem fully consistent with this evidence. However, since we did not include the decay to S${}_{0}$, the comparison cannot be straightforward without a detailed analysis of which transition is actually monitored by the different experiments. This kind of investigation falls outside the scope of the present study, which is mainly focused on the computed spectra. For the case of thymine, where the decay process $\pi \pi^{*}1\to$n${}_{O}\pi^{*}1$ was specifically monitored by time-resolved X-ray spectroscopy [61], our results agree extremely well with experiment (τ= 60 ± 30 fs), predicting a decay constant of 66 fs and 47 fs according to CAM-B3LYP and PBE0 simulations, respectively.

Besides that, we have presented results for dynamics initiated on higher lying bright states, showing that they are all characterized by an ultrafast depopulation of the initially excited state. In forthcoming studies in the condensed phase we shall further investigate this latter topic. Actually, not only in oligonucleotides [98], but also in nucleobases [99,100,101] experiments in the condensed phase show the excited state dynamics can exhibit some dependence on the excitation wavelength.

We believe that the approach here adopted has several interesting characteristics that make it suitable for widespread applications. First, it is based on a straightforward and automated parameterisation of the LVC Hamiltonian, that allows the generation of fully-coupled Hamiltonians for systems with dozen of normal coordinates and several close-lying excited states. The required computational time is only moderately larger than what is necessary for a numerical calculation of the energy gradients of all the involved states. Second, when combined with efficient computation of the QD, this protocol allows to investigate the ultrafast dynamics (and the electronic spectra) involving many coupled states, more than what usually done with on-the-fly semiclassical methods. We selected TD-DFT because, for many classes of systems, when a multireference [102,103] description is not necessary and double-excited states are not involved, it usually represents a good compromise between accuracy and computational cost. LVC Hamiltonians can however be parameterised also with respect to accurate post Hartree–Fock methods like coupled cluster [18], CASSCF [104], different flavours of CASPT2 [19,105] and multireference CIS and CISD [20]. In particular the same maximum-overlap diabatization technique has been recently adopted in combination with RASPT2 to parameterise a LVC Hamiltonian for photoexcited pyrene, a molecule in which TD-DFT switches the correct order of the lowest two excited states [105].

Our approach is best suited for rigid/semi-rigid systems and analysis of dynamics on the ∼100 fs timescale. In the present application, it does not include Duschinsky effects and changes of the vibrational frequencies on the excited state PESs. Extension to this is straightforward by adopting the so called QVC model, at the expense of a more costly dynamics computation [18,19,86]. It should however be warned that the usage of QVC models likely becomes problematic when several coupled states are considered, since it becomes highly probable to find modes with imaginary frequencies (even in a diabatic representation), whose proper description would require an anharmonic treatment. While a general inclusion of anharmonic effects in nonadiabatic calculations of polyatomic molecules remain a big challenge [106,107], limited generalizations of LVC models, including certain vibrational modes at anharmonic level are possible, at the cost of additional effort in parameterising the PES [104].

The present approach is instead not suitable in general to describe nonadiabatic events at conical intersections occurring at very distorted geometries, if the anharmonicity cannot be confined in few well defined coordinates. This is the case of the CI with the ground state in all nucleobases. Possible solutions require the usage of properly defined sets of internal coordinates [104,108].

Even considering the above limitations, a number of possible generalizations of the present approach are possible. For instance, we are working to include in the present scheme the effect of solvent/environment on the dynamics. It is clear that a large proportion of experimental studies are conducted in solution, and the nucleobases studied in this work are found in the DNA/RNA environment. In this respect, we have recently introduced a mixed quantum classical (MQC) scheme, named the adiabatic molecular dynamics generalised vertical Hessian (Ad-MD|gVH) approach, that uses a projection based scheme to partition degrees of freedom into flexible coordinates (of the solute or of the environment, that are treated classically) and stiff coordinates (that are treated quantum mechanically) [48]. Work is ongoing to combine Ad-MD|gVH with nonadiabatic QD approaches, with the scope to extend the kind of calculations presented here to systems with flexible pendants in solution or in a heterogeneous matrix. A first basic implementation of a MQC dynamics for Thy in water, where Thy is described by an LVC Hamiltonian and the solvent is described by classical molecular dynamics was presented in Ref. [109].

## Figures and Tables

**Table 1:** Energies ($E_{i}^{0}$), oscillator strengths $f_{i}$, and electronic characters for the predominant excited states of the pyrimidine bases involved in the dynamics, calculated at the ground-state minimum (FC point, $C_{s}$ symmetry). CAM-B3LYP and PBE0 calculations with 6-311+G(d,p) basis set in gas phase. Energies in eV.

**Uracil**							
**CAM-B3LYP**				**PBE0**			
**State**	$E_{i}^{0}$	$f_{i}$	**Char.**	**State**	$E_{i}^{0}$	$f_{i}$	**Char.**
S${}_{1}$	5.10	0.000	n${}_{O}\pi^{*}$1	S${}_{1}$	4.82	0.000	n${}_{O}\pi^{*}$1
S${}_{2}$	5.50	0.190	$\pi \pi^{*}$1	S${}_{2}$	5.33	0.150	$\pi \pi^{*}$1
S${}_{3}$	6.18	0.003	πRy${}_{\sigma }$1	S${}_{4}$	6.05	0.002	πRy${}_{\sigma }$1
S${}_{5}$	6.62	0.045	$\pi \pi^{*}$2	S${}_{5}$	6.14	0.039	$\pi \pi^{*}$2
S${}_{6}$	6.88	0.170	$\pi \pi^{*}$3	S${}_{7}$	6.63	0.130	$\pi \pi^{*}$3
**Thymine**							
**CAM-B3LYP**				**PBE0**			
**State**	$E_{i}^{0}$	$f_{i}$	**Char.**	**State**	$E_{i}^{0}$	$f_{i}$	**Char.**
S${}_{1}$	5.14	0.000	n${}_{O}\pi^{*}1$	S${}_{1}$	4.89	0.000	n${}_{O}\pi^{*}1$
S${}_{2}$	5.31	0.192	$\pi \pi^{*}1$	S${}_{2}$	5.13	0.154	$\pi \pi^{*}1$
S${}_{3}$	5.94	0.001	$\pi Ry_{\sigma }1$	S${}_{3}$	5.80	0.000	$\pi Ry_{\sigma }1$
S${}_{4}$	6.47	0.000	n${}_{O}\pi^{*}2$	S${}_{4}$	6.10	0.000	n${}_{O}\pi^{*}2$
S${}_{5}$	6.67	0.055	$\pi \pi^{*}2$	S${}_{5}$	6.23	0.071	$\pi \pi^{*}2$
S${}_{6}$	6.73	0.218	$\pi \pi^{*}3$	S${}_{6}$	6.45	0.155	$\pi \pi^{*}3$
**Cytosine**							
**CAM-B3LYP**				**PBE0**			
**State**	$E_{i}^{0}$	$f_{i}$	**Char.**	**State**	$E_{i}^{0}$	$f_{i}$	**Char.**
S${}_{1}$	5.01	0.067	$\pi \pi^{*}1$	S${}_{1}$	4.79	0.047	$\pi \pi^{*}1$
S${}_{2}$	5.29	0.002	n${}_{N}\pi^{*}1$	S${}_{2}$	4.97	0.001	n${}_{N}\pi^{*}1$ + n${}_{O}\pi^{*}1$
S${}_{4}$	5.91	0.000	n${}_{O}\pi^{*}1$	S${}_{3}$	5.36	0.001	n${}_{N}\pi^{*}1$ − n${}_{O}\pi^{*}1$
S${}_{5}$	5.94	0.134	$\pi \pi^{*}2$	S${}_{4}$	5.61	0.099	$\pi \pi^{*}2$
S${}_{6}$	6.13	0.000	n${}_{O}\pi^{*}2$	S${}_{6}$	5.84	0.000	n${}_{O}\pi^{*}2$

**Table 2:** Energies ($E_{i}^{0}$), oscillator strengths $f_{i}$, and electronic characters for the predominant excited states of the purine bases involved in the dynamics, calculated at the ground-state minimum (FC point, $C_{s}$ symmetry). CAM-B3LYP and PBE0 calculations with 6-311+G(d,p) basis set in gas phase. Energies in eV.

**Adenine**							
**CAM-B3LYP**				**PBE0**			
**State**	$E_{i}^{0}$	$f_{i}$	**Char.**	**State**	$E_{i}^{0}$	$f_{i}$	**Char.**
S${}_{1}$	5.37	0.000	n${}_{N}\pi^{*}1$	S${}_{1}$	5.11	0.001	n${}_{N}\pi^{*}1$
S${}_{2}$	5.39	0.286	L${}_{a}$	S${}_{2}$	5.16	0.231	L${}_{a}$
S${}_{3}$	5.52	0.015	L${}_{b}$	S${}_{3}$	5.41	0.037	L${}_{b}$
S${}_{4}$	5.87	0.009	πRy${}_{\sigma }$1	S${}_{4}$	5.65	0.007	πRy${}_{\sigma }$1
**9H-Guanine**							
**CAM-B3LYP**				**PBE0**			
**State**	$E_{i}^{0}$	$f_{i}$	**Char.**	**State**	$E_{i}^{0}$	$f_{i}$	**Char.**
S${}_{1}$	5.18	0.173	L${}_{a}$	S${}_{1}$	4.86	0.002	πRy${}_{\sigma }$1
S${}_{2}$	5.22	0.003	πRy${}_{\sigma }$1	S${}_{2}$	5.04	0.153	L${}_{a}$
S${}_{3}$	5.61	0.000	n${}_{O}\pi^{*}$1	S${}_{3}$	5.36	0.282	L${}_{b}$
S${}_{4}$	5.63	0.336	L${}_{b}$	S${}_{5}$	5.47	0.000	n${}_{O}\pi^{*}$1
**7H-Guanine**							
**CAM-B3LYP**				**PBE0**			
**State**	$E_{i}^{0}$	$f_{i}$	**Char.**	**State**	$E_{i}^{0}$	$f_{i}$	**Char.**
S${}_{1}$	4.91	0.151	L${}_{a}$	S${}_{1}$	4.72	0.127	L${}_{a}$
S${}_{2}$	5.30	0.004	πRy${}_{\sigma }$1	S${}_{2}$	4.98	0.003	πRy${}_{\sigma }$1
S${}_{3}$	5.51	0.000	n${}_{O}\pi^{*}$1 (+n${}_{N9}\pi^{*}$)	S${}_{3}$	5.25	0.000	n${}_{O}\pi^{*}$1 (+n${}_{N9}\pi^{*}$)
S${}_{5}$	5.87	0.158	L${}_{b}$	S${}_{5}$	5.59	0.122	L${}_{b}$

## Data Availability

Data is contained within the article and Supplementary Material.

## Supplementary Materials

The following data are included in the Supplementary Materials file: properties of the adiabatic states. Natural transition orbitals. Energies of the diabatic states minima estimated from the LVC model. Norm of the diabatic coupling vectors. Absorption spectra showing the contribution of the individual states and absolute calculated intensities. Analysis on the effect of imposing planar geometry on Gua. Additional results on dynamics and spectra.
