# Supplementary material for: Nonadiabatic Absorption Spectra and Ultrafast Dynamics of DNA and RNA Photoexcited Nucleobases
Source: Molecules. 2021 Mar 20;26(6):1743. doi: 10.3390/molecules26061743 (PMC8003674; doi:10.3390/molecules26061743)
Supplement: Supplementary file 1 [file molecules-26-01743-s001.pdf]

Supporting Information: Nonadiabatic Absorption Spectra and Ultrafast Dynamics of DNA and RNA Photoexcited Nucleobases.

James A. Green,[1,‡] Martha Yaghoubi Jouybari,[2,‡] Daniel Aranda,[2] Roberto Improta,[1,*] and Fabrizio Santoro[2,*]

‡ These authors contributed equally to this work.

* Email: robimp@unina.it (RI);fabrizio.santoro@pi.iccom.cnr.it (FS)

[1] *CNR–Consiglio Nazionale delle Ricerche, Istituto di Biostrutture e Bioimmagini (IBB-CNR), via Mezzocannone 16, I-80136 Napoli, Italy*

[2] *CNR–Consiglio Nazionale delle Ricerche, Istituto di Chimica dei Composti Organo Metallici (ICCOM-CNR), SS di Pisa, Area della Ricerca, via G. Moruzzi 1, I-56124 Pisa, Italy*

Contents

S1 Electronic Structure and LVC Data **S-3**
S1.1 Uracil S-3
S1.2 Thymine S-9
S1.3 Cytosine S-14
S1.4 Adenine S-19
S1.5 9H-Guanine S-23
S1.5.1 C_s Symmetry S-23
S1.5.2 No Symmetry S-28
S1.6 7H-Guanine S-34

S2 Additional Dynamics and Spectra **S-37**
S2.1 Uracil S-37
S2.2 Thymine S-40
S2.3 Cytosine S-42
S2.4 Adenine S-44
S2.5 9H-Guanine S-46
S2.6 7H-Guanine S-49

S1 Electronic Structure and LVC Data

In this section is presented data from TD-DFT calculations at the FC point for each nucleobase that are used in paramaterising the LVC models, including: excitation energies, oscillator strengths, predominant orbital contributions, assigned characters of the states and natural transition orbitals (NTOs). The energies of the diabatic states in the minima of each state according to the LVC models are also tabulated, as well as the norm of the coupling vectors: $\sqrt{\boldsymbol{\lambda}_{ij} \cdot \boldsymbol{\lambda}_{ij}}$

S1.1 Uracil

Table S1: Energies (E_i^0), oscillator strengths f_i, electronic characters and main contributions in terms of transitions among Kohn-Sham orbitals for the lowest excited states of Uracil used in the LVC model at the ground-state minimum (FC point). CAM-B3LYP and PBE0 calculations with the 6-311+G(d,p) and 6-31G(d) basis sets in gas phase. Energies in eV.

	CAM-B3LYP				PBE0			
State	E_i^0	f_i	Trans.	Char.	E_i^0	f_i	Trans.	Char.
				6-311+G(d,p)				
S_1	5.10	0.0000	H-1→L	$n_O\pi^*1$	4.82	0.0000	H-1→L	$n_O\pi^*1$
S_2	5.50	0.1902	H→L	$\pi\pi^*1$	5.33	0.1496	H→L	$\pi\pi^*1$
S_3	6.18	0.0032	H→L+1	$\pi Ry_\sigma 1$	6.02	0.0001	H-3→L	$n_O\pi^*2$
S_4	6.38	0.0000	H-1→L+3<br>H-3→L+3	$n_O\pi^*2$	6.05	0.0024	H→L+1	$\pi Ry_\sigma 1$
S_5	6.62	0.0452	H-2→L	$\pi\pi^*2$	6.14	0.0392	H-2→L	$\pi\pi^*2$
S_6	6.88	0.1680	H→L+3	$\pi\pi^*3$	6.44	0.0004	H-1→L+2	$n_O\pi^*3$
S_7	7.17	0.0007	H→L+2	$\pi Ry_\sigma 2$	6.63	0.1263	H→L+2	$\pi\pi^*3$
S_8	7.17	0.0002	H-3→L	$n_O\pi^*3$				
				6-31G(d)				
S_1	5.09	0.0001	H-1→L	$n_O\pi^*1$	4.82	0.0001	H-1→L	$n_O\pi^*1$
S_2	5.65	0.1724	H→L	$\pi\pi^*1$	5.46	0.1336	H→L	$\pi\pi^*1$
S_3	6.40	0.0000	H-1→L+1<br>H-3→L+1	$n_O\pi^*2$	6.05	0.0000	H-3→L	$n_O\pi^*2$
S_4	6.73	0.0444	H-2→L	$\pi\pi^*2$	6.22	0.0397	H-2→L	$\pi\pi^*2$
S_5	7.18	0.1395	H→L+1	$\pi\pi^*3$	6.49	0.0000	H-1→L+1	$n_O\pi^*3$
S_6	7.23	0.0000	H-3→L	$n_O\pi^*3$	6.89	0.1170	H→L+1	$\pi\pi^*3$

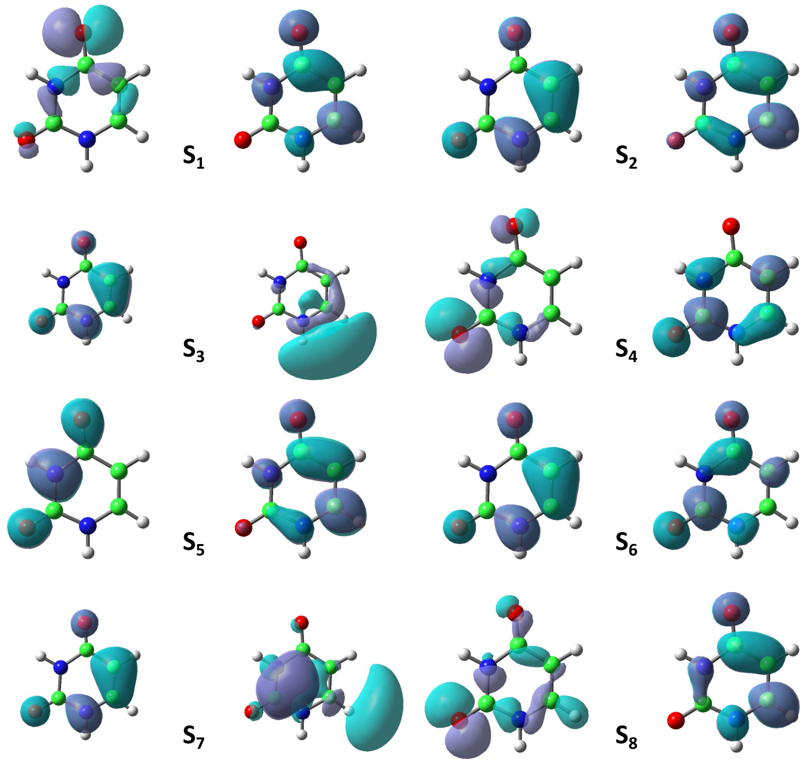

Figure S1: NTOs of Uracil in gas phase at ground state geometry using the CAM-B3LYP functional and 6-311+G(d,p) basis with an isovalue 0.04 for all orbitals, except the virtual NTOs of the rydberg states which use an isovalue of 0.03.

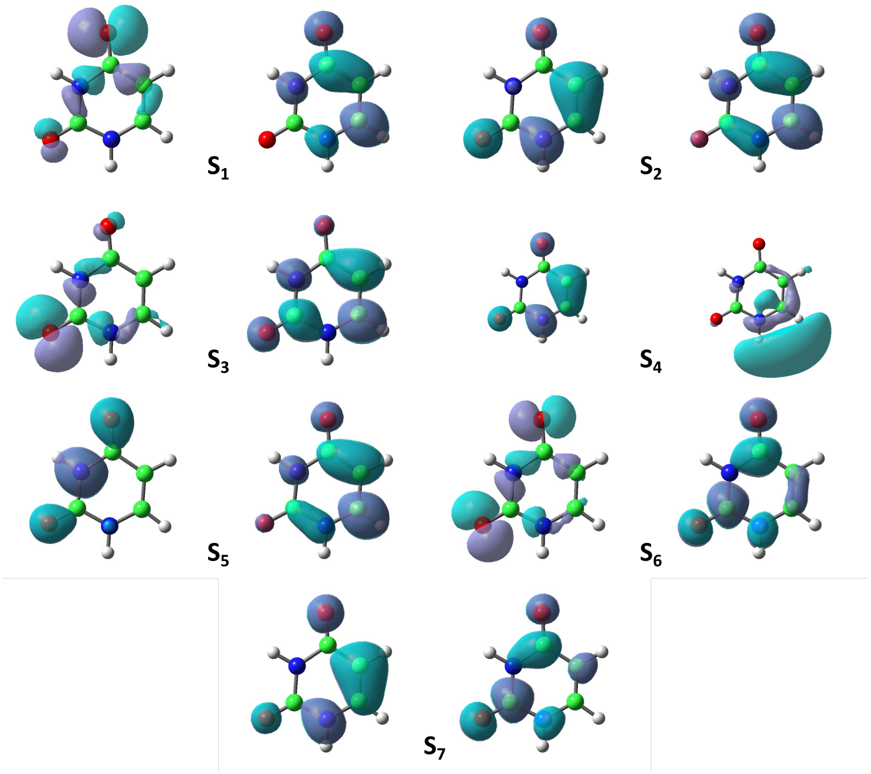

Figure S2: NTOs of Uracil in gas phase at ground state geometry using the PBE0 functional and 6-311+G(d,p) basis with an isovalue 0.04 for all orbitals, except the virtual NTOs of the rydberg states which use an isovalue of 0.03.

Table S2: Energies of the diabatic states at the FC point and in the minima of each state according to the LVC model for Uracil in C_s symmetry in the gas phase with CAM-B3LYP and PBE0 6-311+G(d,p) calculations. N.b. 2nd Rydberg state not included in PBE0 LVC potential, as it is higher in energy.

	$n_O\pi^*1$	$\pi\pi^*1$	$\pi Ry_\sigma 1$	$n_O\pi^*2$	$\pi\pi^*2$	$\pi\pi^*3$	$\pi Ry_\sigma 2$	$n_O\pi^*3$
IN MIN ↓	CAM-B3LYP							
S_0	5.099	5.501	6.178	6.378	6.616	6.881	7.165	7.173
$n_O\pi^*1$	4.667	5.568	6.727	6.641	6.273	7.375	7.584	7.283
$\pi\pi^*1$	5.084	5.151	6.321	6.491	6.419	6.975	7.221	7.141
$\pi Ry_\sigma 1$	5.435	5.512	5.959	6.584	7.045	6.723	7.128	7.406
$n_O\pi^*2$	5.452	5.785	6.686	5.857	6.738	7.128	7.591	7.290
$\pi\pi^*2$	5.014	5.643	7.077	6.668	5.927	8.045	7.919	7.410
$\pi\pi^*3$	5.647	5.730	6.287	6.590	7.576	6.395	7.304	7.589
$\pi Ry_\sigma 2$	5.219	5.340	6.055	6.415	6.814	6.667	7.032	7.174
$n_O\pi^*3$	4.997	5.339	6.412	6.195	6.384	7.032	7.253	6.953
	PBE0							
S_0	4.821	5.332	6.053	6.019	6.135	6.625	-	6.438
$n_O\pi^*1$	4.317	5.401	6.637	6.028	5.726	7.178	-	6.506
$\pi\pi^*1$	4.687	5.030	6.219	5.826	5.858	6.767	-	6.457
$\pi Ry_\sigma 1$	5.134	5.429	5.820	6.306	6.497	6.520	-	6.713
$n_O\pi^*2$	4.859	5.371	6.640	5.485	5.873	7.176	-	6.353
$\pi\pi^*2$	4.538	5.384	6.812	5.853	5.505	7.523	-	6.640
$\pi\pi^*3$	5.254	5.557	6.099	6.420	6.787	6.241	-	6.607
$\pi Ry_\sigma 2$	-	-	-	-	-	-	-	-
$n_O\pi^*3$	4.655	5.320	6.366	5.671	5.977	6.681	-	6.167

Table S3: Energies of the diabatic states at the FC point and in the minima of each state according to the LVC model for Uracil in C_s symmetry in the gas phase with CAM-B3LYP and PBE0 6-31G(d) calculations.

	$n_O\pi^*1$	$\pi\pi^*1$	$n_O\pi^*2$	$\pi\pi^*2$	$\pi\pi^*3$	$n_O\pi^*3$
IN MIN ↓	CAM-B3LYP					
S_0	5.095	5.649	6.405	6.726	7.180	7.227
$n_O\pi^*1$	4.631	5.725	6.689	6.378	7.718	7.177
$\pi\pi^*1$	5.062	5.294	6.497	6.467	7.320	7.073
$n_O\pi^*2$	5.475	5.946	5.844	6.847	7.446	7.154
$\pi\pi^*2$	5.012	5.763	6.694	5.997	8.431	7.113
$\pi\pi^*3$	5.739	6.004	6.681	7.818	6.610	7.981
$n_O\pi^*3$	5.154	5.713	6.344	6.456	7.937	6.654
	PBE0					
S_0	4.825	5.460	6.048	6.223	6.886	6.490
$n_O\pi^*1$	4.287	5.536	6.053	5.791	7.519	6.573
$\pi\pi^*1$	4.681	5.143	5.802	5.906	7.080	6.488
$n_O\pi^*2$	4.873	5.477	5.468	5.948	7.542	6.385
$\pi\pi^*2$	4.521	5.492	5.859	5.557	7.872	6.691
$\pi\pi^*3$	5.426	5.842	6.630	7.049	6.380	6.777
$n_O\pi^*3$	4.668	5.438	5.661	6.056	6.965	6.192

Table S4: Norm of the coupling vectors for the LVC model in the gas phase at the FC point of Uracil in C_s symmetry obtained by diabatisation at the CAM-B3LYP and PBE0/6-311+G(d,p) level. N.b. 2nd Rydberg state not included in PBE0 LVC potential, as it is higher in energy.

STATE	$n_O\pi^*1$	$\pi\pi^*1$	$\pi Ry_\sigma 1$	$n_O\pi^*2$	$\pi\pi^*2$	$\pi\pi^*3$	$\pi Ry_\sigma 2$	$n_O\pi^*3$
	CAM-B3LYP							
$n_O\pi^*1$	0.430							
$\pi\pi^*1$	0.050	0.347						
$\pi Ry_\sigma 1$	0.004	0.091	0.307					
$n_O\pi^*2$	0.125	0.043	0.012	0.471				
$\pi\pi^*2$	0.085	0.126	0.014	0.034	0.493			
$\pi\pi^*3$	0.032	0.171	0.113	0.061	0.063	0.397		
$\pi Ry_\sigma 2$	0.058	0.120	0.087	0.091	0.044	0.160	0.213	
$n_O\pi^*3$	0.109	0.110	0.061	0.173	0.091	0.121	0.213	0.293
	PBE0							
$n_O\pi^*1$	0.459							
$\pi\pi^*1$	0.051	0.318						
$\pi Ry_\sigma 1$	0.016	0.086	0.328					
$n_O\pi^*2$	0.181	0.062	0.045	0.476				
$\pi\pi^*2$	0.081	0.178	0.032	0.061	0.476			
$\pi\pi^*3$	0.039	0.149	0.106	0.042	0.144	0.353		
$\pi Ry_\sigma 2$	-	-	-	-	-	-	-	
$n_O\pi^*3$	0.170	0.067	0.019	0.262	0.078	0.084	-	0.341

Table S5: Norm of the coupling vectors for the LVC model in the gas phase at the FC point of Uracil in C_s symmetry obtained by diabatisation at the CAM-B3LYP and PBE0/6-31G(d) level.

STATE	$n_O\pi^*1$	$\pi\pi^*1$	$n_O\pi^*2$	$\pi\pi^*2$	$\pi\pi^*3$	$n_O\pi^*3$
	CAM-B3LYP					
$n_O\pi^*1$	0.451					
$\pi\pi^*1$	0.052	0.353				
$n_O\pi^*2$	0.126	0.042	0.493			
$\pi\pi^*2$	0.084	0.145	0.035	0.511		
$\pi\pi^*3$	0.041	0.177	0.059	0.107	0.429	
$n_O\pi^*3$	0.128	0.097	0.217	0.098	0.086	0.479
	PBE0					
$n_O\pi^*1$	0.478					
$\pi\pi^*1$	0.053	0.330				
$n_O\pi^*2$	0.185	0.064	0.501			
$\pi\pi^*2$	0.080	0.195	0.061	0.493		
$\pi\pi^*3$	0.050	0.163	0.037	0.173	0.401	
$n_O\pi^*3$	0.176	0.061	0.276	0.077	0.094	0.362

S1.2 Thymine

Table S6: Energies (E_i^0), electronic characters and main contributions in terms of transitions among Kohn-Sham orbitals for the lowest excited states of of Thymine at the ground-state minimum (FC point). CAM-B3LYP and PBE0 calculations with 6-311+G(d,p) and 6-31G(d) basis sets in gas phase. Energies in eV.

	CAM-B3LYP				PBE0			
State	E_i^0	f_i	Trans.	Char.	E_i^0	f_i	Trans.	Char.
				6-311+G(d,p)				
S_1	5.14	0.0000	H-1→ L	$n_O\pi^*1$	4.89	0.0000	H-1→ L	$n_O\pi^*1$
S_2	5.31	0.1915	H→ L	$\pi\pi^*1$	5.13	0.1541	H→ L	$\pi\pi^*1$
S_3	5.94	0.0006	H→ L+1	$\pi Ry_\sigma 1$	5.80	0.0004	H→ L+1	$\pi Ry_\sigma 1$
			H-2→ L					
S_4	6.47	0.0000	H-1→ L+4	$n_O\pi^*2$	6.10	0.0001	H-3→ L	$n_O\pi^*2$
			H-3→ L+4				H-1→ L+2	
			H-3→ L					
S_5	6.67	0.0550	H-2→ L	$\pi\pi^*2$	6.23	0.0711	H-2→ L	$\pi\pi^*2$
S_6	6.73	0.2182	H→ L+4	$\pi\pi^*3$	6.45	0.1552	H→ L+2	$\pi\pi^*3$
S_7	6.78	0.0013	H→ L+3	$\pi Ry_\sigma 2$	6.48	0.0003	H-1→ L+2	$n_O\pi^*3$
							H-3→ L	
				6-31G(d)				
S_1	5.15	0.0001	H-1→ L	$n_O\pi^*1$	4.91	0.0001	H-1→ L	$n_O\pi^*1$
S_2	5.51	0.1809	H→ L	$\pi\pi^*1$	5.31	0.1427	H→ L	$\pi\pi^*1$
S_3	6.48	0.0000	H-1→ L+1	$n_O\pi^*2$	6.14	0.0000	H-3→ L	$n_O\pi^*2$
			H-3→ L				H-1→ L+1	
S_4	6.83	0.0768	H-2→ L	$\pi\pi^*2$	6.35	0.0726	H-2→ L	$\pi\pi^*2$
S_5	6.96	0.1736	H→ L+1	$n_O\pi^*3$	6.52	0.0000	H-1→ L+1	$n_O\pi^*3$
S_6	7.23	0.0000	H-3→ L	$\pi\pi^*3$	6.66	0.1395	H→ L+1	$\pi\pi^*3$

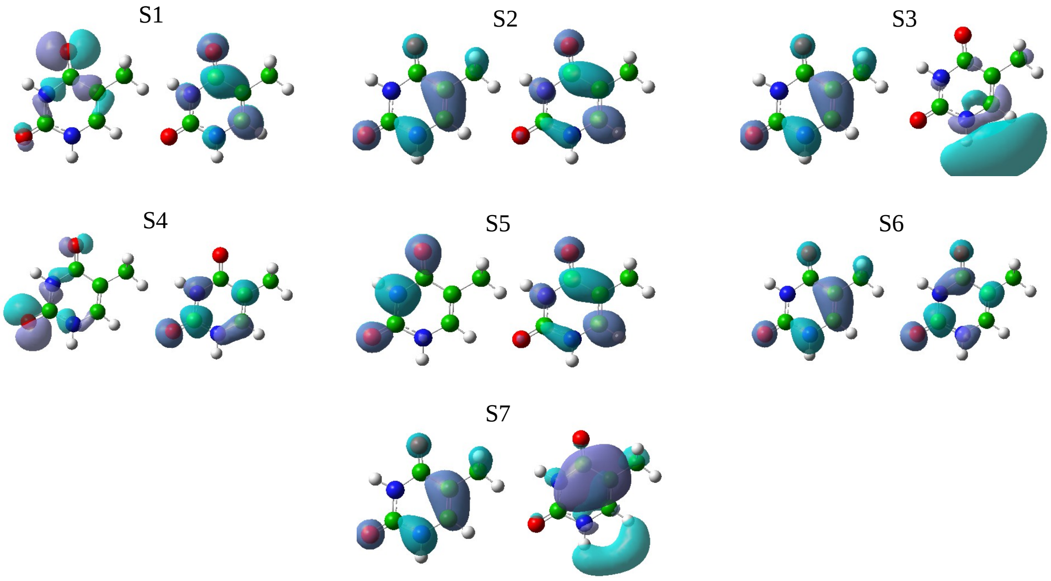

Figure S3: NTOs of Thymine in gas phase at ground state geometry using the CAM-B3LYP functional and 6-311+G(d,p) basis with an isovalue 0.04 for all orbitals, except the virtual NTOs of the rydberg states which use an isovalue of 0.03.

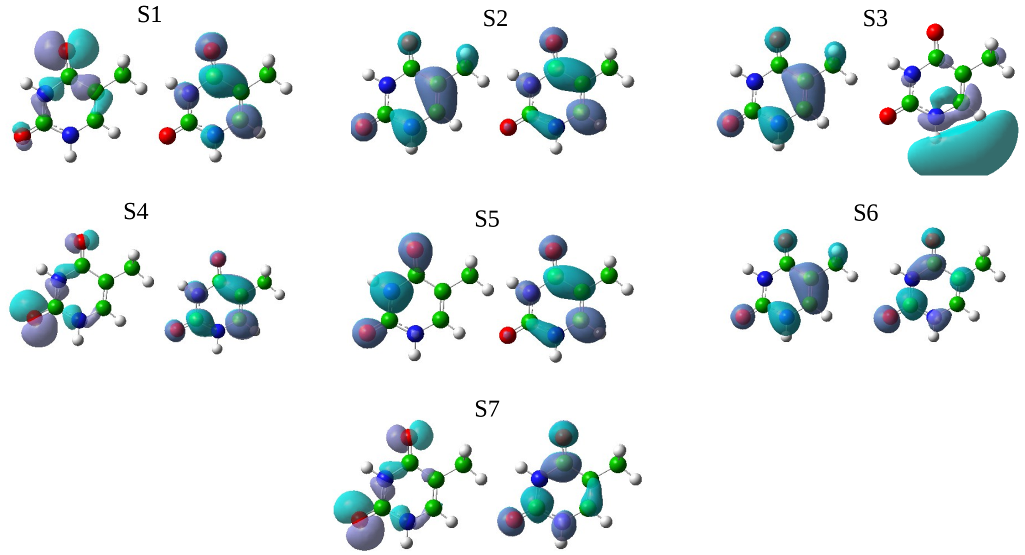

Figure S4: NTOs of Thymine in gas phase at ground state geometry using the PBE0 functional and 6-311+G(d,p) basis with an isovalue 0.04 for all orbitals, except the virtual NTOs of the rydberg states which use an isovalue of 0.03.

Table S7: Energies of the diabatic states at the FC point and in the minima of each state according to the LVC model for Thymine in C_s symmetry in the gas phase with CAM-B3LYP and PBE0 6-311+G(d,p) calculations.

	$n_O\pi^*1$	$\pi\pi^*1$	$\pi Ry_\sigma 1$	$n_O\pi^*2$	$\pi\pi^*2$	$\pi\pi^*3$	$n_O\pi^*3$
IN MIN ↓	CAM-B3LYP						
S_0	5.141	5.312	5.943	6.466	6.674	6.729	6.777
$n_O\pi^*1$	4.735	5.380	6.481	6.732	6.332	7.234	7.338
$\pi\pi^*1$	5.180	4.936	6.051	6.636	6.532	6.811	6.840
$\pi Ry_\sigma 1$	5.525	5.294	5.692	6.695	7.126	6.555	6.558
$n_O\pi^*2$	5.545	5.650	6.466	5.922	6.894	6.923	7.337
$\pi\pi^*2$	5.029	5.430	6.780	6.778	6.038	7.802	7.665
$\pi\pi^*3$	5.741	5.518	6.019	6.617	7.612	6.228	6.809
$\pi Ry_\sigma 2$	5.548	5.251	5.725	6.734	7.178	6.512	6.525
	$n_O\pi^*1$	$\pi\pi^*1$	$\pi Ry_\sigma 1$	$n_O\pi^*2$	$\pi\pi^*2$	$\pi\pi^*3$	$n_O\pi^*3$
IN MIN ↓	PBE0						
S_0	4.893	5.133	5.801	6.100	6.228	6.455	6.481
$n_O\pi^*1$	4.420	5.212	6.381	6.052	5.840	7.031	6.615
$\pi\pi^*1$	4.821	4.810	5.925	5.908	6.007	6.557	6.555
$\pi Ry_\sigma 1$	5.266	5.200	5.534	6.438	6.597	6.342	6.830
$n_O\pi^*2$	4.968	5.213	6.468	5.504	5.993	7.096	6.394
$\pi\pi^*2$	4.606	5.163	6.478	5.844	5.653	7.246	6.679
$\pi\pi^*3$	5.409	5.324	5.833	6.558	6.857	6.042	6.676
$n_O\pi^*3$	4.861	5.191	6.191	5.725	6.159	6.545	6.173

Table S8: Energies of the diabatic states at the FC point and in the minima of each state according to the LVC model for Thymine in C_s symmetry in the gas phase with CAM-B3LYP and PBE0 6-31G(d) calculations.

	$n_O\pi^*1$	$\pi\pi^*1$	$n_O\pi^*2$	$\pi\pi^*2$	$n_O\pi^*3$	$\pi\pi^*3$
IN MIN ↓	CAM-B3LYP					
S_0	5.150	5.514	6.482	6.829	6.960	7.233
$n_O\pi^*1$	4.712	5.586	6.777	6.477	7.493	7.143
$\pi\pi^*1$	5.165	5.133	6.636	6.603	7.081	7.050
$n_O\pi^*2$	5.588	5.867	5.901	7.031	7.195	7.181
$\pi\pi^*2$	5.049	5.596	6.793	6.140	8.088	7.037
$n_O\pi^*3$	5.797	5.805	6.687	7.818	6.409	8.020
$\pi\pi^*3$	5.331	5.659	6.558	6.653	7.906	6.524
	$n_O\pi^*1$	$\pi\pi^*1$	$n_O\pi^*2$	$\pi\pi^*2$	$n_O\pi^*3$	$\pi\pi^*3$
IN MIN ↓	PBE0					
S_0	4.913	5.311	6.135	6.348	6.517	6.656
$n_O\pi^*1$	4.408	5.392	6.114	5.951	6.630	7.261
$\pi\pi^*1$	4.825	4.975	5.916	6.092	6.525	6.801
$n_O\pi^*2$	4.990	5.359	5.532	6.104	6.359	7.300
$\pi\pi^*2$	4.612	5.320	5.888	5.747	6.670	7.434
$n_O\pi^*3$	4.865	5.326	5.717	6.244	6.174	6.824
$\pi\pi^*3$	5.458	5.565	6.621	6.970	6.787	6.211

Table S9: Norm of the coupling vectors for the LVC model in the gas phase at the FC point of Thymine in C_s symmetry obtained by diabatisation at the CAM-B3LYP and PBE0/6-311+G(d,p) level.

STATE	$n_O\pi^*1$	$\pi\pi^*1$	$\pi Ry_\sigma 1$	$n_O\pi^*2$	$\pi\pi^*2$	$\pi\pi^*3$	$\pi Ry_\sigma 2$
	CAM-B3LYP						
$n_O\pi^*1$	0.416						
$\pi\pi^*1$	0.059	0.358					
$\pi Ry_\sigma 1$	0.003	0.076	0.322				
$n_O\pi^*2$	0.114	0.040	0.008	0.478			
$\pi\pi^*2$	0.080	0.109	0.010	0.025	0.476		
$\pi\pi^*3$	0.039	0.176	0.132	0.063	0.078	0.406	
$\pi Ry_\sigma 2$	0.005	0.189	0.145	0.006	0.021	0.181	0.300
STATE	$n_O\pi^*1$	$\pi\pi^*1$	$\pi Ry_\sigma 1$	$n_O\pi^*2$	$\pi\pi^*2$	$\pi\pi^*3$	$n_O\pi^*3$
	PBE0						
$n_O\pi^*1$	0.444						
$\pi\pi^*1$	0.058	0.326					
$\pi Ry_\sigma 1$	0.004	0.065	0.343				
$n_O\pi^*2$	0.179	0.076	0.007	0.500			
$\pi\pi^*2$	0.077	0.160	0.041	0.062	0.457		
$\pi\pi^*3$	0.037	0.158	0.118	0.041	0.181	0.366	
$n_O\pi^*3$	0.152	0.065	0.012	0.250	0.068	0.085	0.363

Table S10: Norm of the coupling vectors for the LVC model in the gas phase at the FC point of Thymine in C_s symmetry obtained by diabatisation at the CAM-B3LYP and PBE0/6-31G(d) level.

STATE	$n_O\pi^*1$	$\pi\pi^*1$	$n_O\pi^*2$	$\pi\pi^*2$	$n_O\pi^*3$	$\pi\pi^*3$
	CAM-B3LYP					
$n_O\pi^*1$	0.437					
$\pi\pi^*1$	0.060	0.363				
$n_O\pi^*2$	0.114	0.036	0.499			
$\pi\pi^*2$	0.081	0.134	0.031	0.499		
$n_O\pi^*3$	0.042	0.178	0.060	0.127	0.427	
$\pi\pi^*3$	0.128	0.098	0.213	0.091	0.082	0.531
STATE	$n_O\pi^*1$	$\pi\pi^*1$	$n_O\pi^*2$	$\pi\pi^*2$	$n_O\pi^*3$	$\pi\pi^*3$
	PBE0					
$n_O\pi^*1$	0.463					
$\pi\pi^*1$	0.059	0.335				
$n_O\pi^*2$	0.183	0.068	0.509			
$\pi\pi^*2$	0.075	0.179	0.058	0.470		
$n_O\pi^*3$	0.158	0.065	0.272	0.069	0.389	
$\pi\pi^*3$	0.048	0.162	0.040	0.217	0.091	0.380

S1.3 Cytosine

Table S11: Energies (E_i^0), electronic characters and main contributions in terms of transitions among Kohn-Sham orbitals for the lowest excited states of of Cytosine at the ground-state minimum (FC point). CAM-B3LYP and PBE0 calculations with 6-311+G(d,p) and 6-31G(d) basis sets in gas phase. Energies in eV.

	CAM-B3LYP				PBE0			
State	E_i^0	f_i	Trans.	Char.	E_i^0	f_i	Trans.	Char.
				6-311+G(d,p)				
S_1	5.01	0.0671	H→ L	$\pi\pi^*1$	4.79	0.0468	H→ L	$\pi\pi^*1$
S_2	5.29	0.0021	H-3→ L	$n_N\pi^*1$	4.97	0.0014	H-1→ L	$n_N\pi^*1+n_O\pi^*1$
S_3	5.81	0.0038	H→ L+1	$\pi Ry_\sigma 1$	5.36	0.0006	H-3→ L	$n_N\pi^*1-n_O\pi^*1$
S_4	5.91	0.0004	H-2→ L+4	$n_O\pi^*1$	5.61	0.0989	H-2→ L	$\pi\pi^*2$
			H-3→ L					
			H-2→ L					
S_5	5.94	0.134	H-1→ L	$\pi\pi^*2$	5.62	0.0045	H→ L+1	$\pi Ry_\sigma 1$
S_6	6.13	0.0000	H-2→ L+4	$n_O\pi^*2$	5.84	0.0000	H-1→ L+3	$n_O\pi^*2$
			H-2→ L					
S_7	6.34	0.0042	H-1→ L+1	$\pi Ry_\sigma 2$	6.11	0.0020	H→ L+2	$\pi Ry_\sigma 2$
			H-1→ L+2					
				6-31G(d)				
S_1	5.12	0.0578	H→ L	$\pi\pi^*1$	4.88	0.0392	H→ L	$\pi\pi^*1$
S_2	5.34	0.0011	H-3→ L	$n_N\pi^*1$	5.0	0.0004	H-1→ L	$n_N\pi^*1+n_O\pi^*1$
			H-2→ L					
S_3	5.94	0.0006	H-2→ L+1	$n_O\pi^*1$	5.40	0.0012	H-3→ L	$n_N\pi^*1-n_O\pi^*1$
			H-3→ L					
			H-2→ L					
S_4	6.12	0.1354	H-2→ L	$\pi\pi^*2$	5.76	0.1017	H-2→ L	$\pi\pi^*2$
S_5	6.17	0.0000	H-2→ L+1	$n_O\pi^*2$	5.90	0.0001	H-1→ L+1	$n_N\pi^*2+n_O\pi^*2$
			H-2→ L					
S_6	6.91	0.0011	H-3→ L+1	$n_N\pi^*2$	6.66	0.0006	H-3→ L+1	$n_N\pi^*2-n_O\pi^*2$

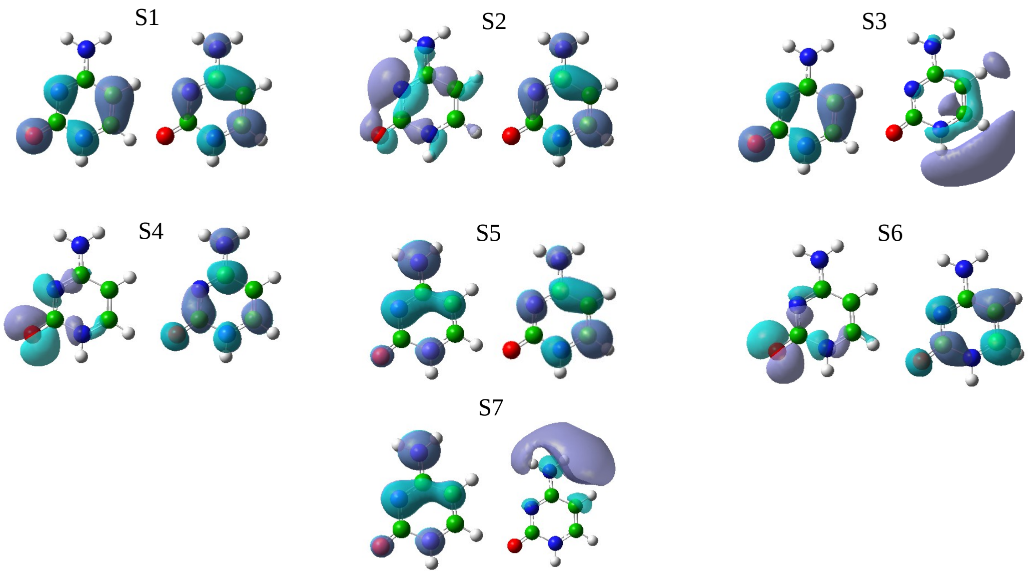

Figure S5: NTOs of Cytosine in gas phase at ground state geometry using the CAM-B3LYP functional and 6-311+G(d,p) basis with an isovalue 0.04 for all orbitals, except the virtual NTOs of the rydberg states which use an isovalue of 0.03

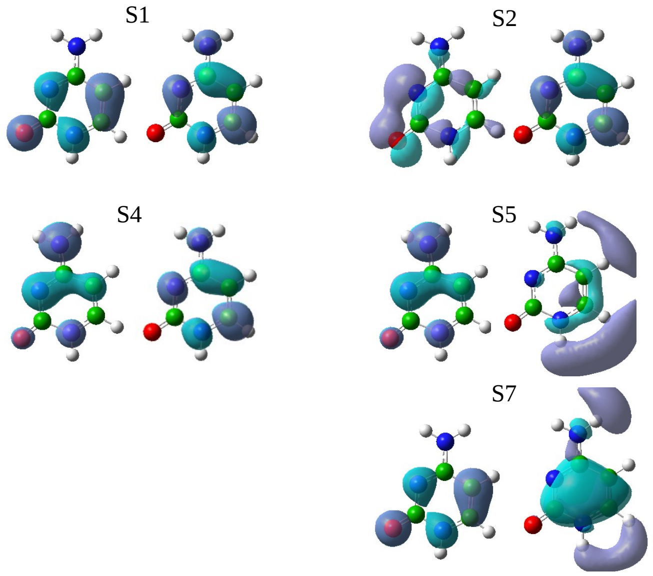

Figure S6: NTOs of Cytosine in gas phase at ground state geometry using the PBE0 functional and 6-311+G(d,p) basis with an isovalue 0.04 for all orbitals, except the virtual NTOs of the rydberg states which use an isovalue of 0.03

Table S12: Energies of the diabatic states at the FC point and in the minima of each state according to the LVC model for Cytosine in C_s symmetry in the gas phase with CAM-B3LYP and PBE0 6-311+G(d,p) calculations.

	$\pi\pi^*1$	$n_N\pi^*1$	$\pi Ry_\sigma 1$	$n_O\pi^*1$	$\pi\pi^*2$	$n_O\pi^*2$	$\pi Ry_\sigma 2$
IN MIN ↓	CAM-B3LYP						
S_0	5.014	5.286	5.809	5.912	5.939	6.133	6.343
$\pi\pi^*1$	4.577	5.389	6.082	5.697	5.794	5.972	6.747
$n_N\pi^*1$	5.209	4.757	6.471	6.235	6.120	6.681	6.896
$\pi Ry_\sigma 1$	4.991	5.559	5.669	6.020	6.147	6.181	6.448
$n_O\pi^*1$	5.118	5.834	6.532	5.157	6.500	6.014	7.236
$\pi\pi^*2$	4.825	5.330	6.269	6.110	5.547	6.077	6.605
$n_O\pi^*2$	4.978	5.866	6.278	5.599	6.053	5.571	6.885
$\pi Ry_\sigma 2$	5.047	5.375	5.839	6.115	5.874	6.179	6.278
	$\pi\pi^*1$	$n_N\pi^*$+$n_O\pi^*1$	$n_N\pi^*$-$n_O\pi^*1$	$\pi\pi^*2$	$\pi Ry_\sigma 1$	$n_O\pi^*2$	$\pi Ry_\sigma 2$
IN MIN ↓	PBE0						
S_0	4.797	4.971	5.356	5.610	5.624	5.843	6.105
$\pi\pi^*1$	4.309	4.817	4.752	5.529	5.931	5.984	6.333
$n_N\pi^*$+$n_O\pi^*1$	4.553	4.573	4.883	5.542	6.063	6.162	6.459
$n_N\pi^*$-$n_O\pi^*1$	4.668	5.064	4.393	6.078	6.441	6.261	6.782
$\pi\pi^*2$	4.623	4.900	5.255	5.215	6.060	6.251	6.522
$\pi Ry_\sigma 1$	4.755	5.151	5.349	5.790	5.485	5.865	5.972
$n_O\pi^*2$	4.978	5.421	5.338	6.151	6.036	5.315	6.479
$\pi Ry_\sigma 2$	4.707	5.097	5.240	5.802	5.522	5.859	5.935

Table S13: Energies of the diabatic states at the FC point and in the minima of each state according to the LVC model for Cytosine in C_s symmetry in the gas phase with CAM-B3LYP and PBE0 6-31G(d) calculations.

STATE	$\pi\pi^*1$	$n_N\pi^*1$	$n_O\pi^*1$	$\pi\pi^*2$	$n_O\pi^*2$	$n_N\pi^*2$
			CAM-B3LYP			
S_0	5.119	5.339	5.943	6.118	6.169	6.912
$\pi\pi^*1$	4.641	5.440	5.662	5.947	5.957	7.516
$n_N\pi^*1$	5.275	4.805	6.198	6.272	6.699	6.835
$n_O\pi^*1$	5.181	5.881	5.123	6.706	6.021	7.556
$\pi\pi^*2$	4.923	5.412	6.164	5.665	6.101	7.507
$n_O\pi^*2$	5.058	5.964	5.603	6.226	5.540	7.449
$n_N\pi^*2$	5.958	5.441	6.479	6.973	6.790	6.199
STATE	$\pi\pi^*1$	$n_N\pi^*1+n_O\pi^*1$	$n_N\pi^*1-n_O\pi^*1$	$\pi\pi^*2$	$n_N\pi^*2+n_O\pi^*2$	$n_N\pi^*2$-$n_O\pi^*2$
			PBE0			
S_0	4.883	5.003	5.403	5.755	5.903	6.657
$\pi\pi^*1$	4.336	4.764	4.808	5.684	6.036	7.411
$n_N\pi^*1+n_O\pi^*1$	4.534	4.566	4.851	5.670	6.166	7.005
$n_N\pi^*1-n_O\pi^*1$	4.699	4.972	4.445	6.207	6.348	7.174
$\pi\pi^*2$	4.703	4.919	5.335	5.317	6.337	7.290
$n_N\pi^*2+n_O\pi^*2$	5.055	5.414	5.475	6.336	5.317	6.980
$n_N\pi^*2$-$n_O\pi^*2$	5.825	5.650	5.697	6.685	6.376	5.922

Table S14: Norm of the coupling vectors for the LVC model in the gas phase at the FC point of Cytosine in C_s symmetry obtained by diabatisation at the CAM-B3LYP and PBE0/6-311+G(d,p) level.

STATE	$\pi\pi^*1$	$n_N\pi^*1$	$\pi Ry_\sigma 1$	$n_O\pi^*1$	$\pi\pi^*2$	$n_O\pi^*2$	$\pi Ry_\sigma 2$
	CAM-B3LYP						
$\pi\pi^*1$	0.386						
$n_N\pi^*1$	0.130	0.361					
$\pi Ry_\sigma 1$	0.073	0.018	0.231				
$n_O\pi^*1$	0.097	0.143	0.071	0.555			
$\pi\pi^*2$	0.163	0.147	0.030	0.076	0.361		
$n_O\pi^*2$	0.076	0.145	0.034	0.285	0.063	0.472	
$\pi Ry_\sigma 2$	0.032	0.013	0.094	0.024	0.080	0.012	0.208
STATE	$\pi\pi^*1$	$n_N\pi^*1+n_O\pi^*1$	$n_N\pi^*1-n_O\pi^*1$	$\pi\pi^*2$	$\pi Ry_\sigma 1$	$n_O\pi^*2$	$\pi Ry_\sigma 2$
	PBE0						
$\pi\pi^*1$	0.420						
$n_N\pi^*1+n_O\pi^*1$	0.105	0.356					
$n_N\pi^*1-n_O\pi^*1$	0.120	0.317	0.572				
$\pi\pi^*2$	0.161	0.134	0.101	0.360			
$\pi Ry_\sigma 1$	0.084	0.014	0.023	0.031	0.234		
$n_O\pi^*2$	0.020	0.178	0.125	0.018	0.000	0.472	
$\pi Ry_\sigma 2$	0.090	0.010	0.008	0.049	0.130	0.008	0.262

Table S15: Norm of the coupling vectors for the LVC model in the gas phase at the FC point of Cytosine in C_s symmetry obtained by diabatisation at the CAM-B3LYP and PBE0/6-31G(d) level.

STATE	$\pi\pi^*1$	$n_N\pi^*1$	$n_O\pi^*1$	$\pi\pi^*2$	$n_O\pi^*2$	$n_N\pi^*2$
	CAM-B3LYP					
$\pi\pi^*1$	0.411					
$n_N\pi^*1$	0.130	0.367				
$n_O\pi^*1$	0.092	0.161	0.583			
$\pi\pi^*2$	0.172	0.126	0.073	0.390		
$n_O\pi^*2$	0.073	0.155	0.300	0.063	0.508	
$n_N\pi^*2$	0.034	0.210	0.149	0.067	0.000	0.401
STATE	$\pi\pi^*1$	$n_N\pi^*1+n_O\pi^*1$	$n_N\pi^*1-n_O\pi^*1$	$\pi\pi^*2$	$n_N\pi^*2+n_O\pi^*2$	$n_N\pi^*2$-$n_O\pi^*2$
	PBE0					
$\pi\pi^*1$	0.451					
$n_N\pi^*1+n_O\pi^*1$	0.102	0.386				
$n_N\pi^*1-n_O\pi^*1$	0.124	0.342	0.565			
$\pi\pi^*2$	0.164	0.118	0.103	0.382		
$n_N\pi^*2+n_O\pi^*2$	0.020	0.192	0.124	0.020	0.504	
$n_N\pi^*2$-$n_O\pi^*2$	0.026	0.136	0.209	0.064	0.000	0.399

S1.4 Adenine

Table S16: Energies (E_i^0), electronic characters and main contributions in terms of transitions among Kohn-Sham orbitals for the lowest excited states of of Adenine at the ground-state minimum (FC point). CAM-B3LYP and PBE0 calculations with 6-311+G(d,p) and 6-31G(d) basis sets in gas phase. Energies in eV.

	CAM-B3LYP				PBE0			
State	E_i^0	f_i	Trans.	Char.	E_i^0	f_i	Trans.	Char.
				6-311+G(d,p)				
S_1	5.37	0.0000	H-1→ L	$n_N\pi^*1$	5.11	0.0005	H-1→ L	$n_N\pi^*1$
S_2	5.39	0.2856	H→ L	L_a	5.16	0.2313	H→ L	L_a
S_3	5.52	0.015	H→ L+3	L_b	5.41	0.0369	H→ L+1	L_b
			H-2→ L				H-2→ L	
S_4	5.87	0.0085	H→ L+1	$\pi Ry_\sigma 1$	5.65	0.0069	H→ L+2	$\pi Ry_\sigma 1$
			H→ L+2					
S_5	5.95	0.0033	H-1→ L+3	$n_N\pi^*2$	5.73	0.0019	H-1→ L+1	$n_N\pi^*2$
S_6	6.21	0.0014	H→ L+2	$\pi Ry_\sigma 2$	5.93	0.0028	H→ L+3	$\pi Ry_\sigma 2$
			H→ L+1					
S_7	6.31	0.001	H-3→ L	$n_N\pi^*3$	6.06	0.0005	H-3→ L	$n_N\pi^*3$
				6-31G(d)				
S_1	5.44	0.0003	H-1→ L	$n_N\pi^*1$	5.18	0.0002	H-1→ L	$n_N\pi^*1$
S_2	5.57	0.2013	H→ L	L_a	5.32	0.1883	H→ L	L_a
S_3	5.64	0.0822	H→ L+1	L_b	5.52	0.0642	H→ L+1	L_b
			H-2→ L				H-2→ L	
S_4	6.03	0.0012	H-1→ L+1	$n_N\pi^*2$	5.80	0.0009	H-1→ L+1	$n_N\pi^*2$
S_5	6.42	0.0007	H-3→ L	$n_N\pi^*3$	6.17	0.0003	H-3→ L	$n_N\pi^*3$

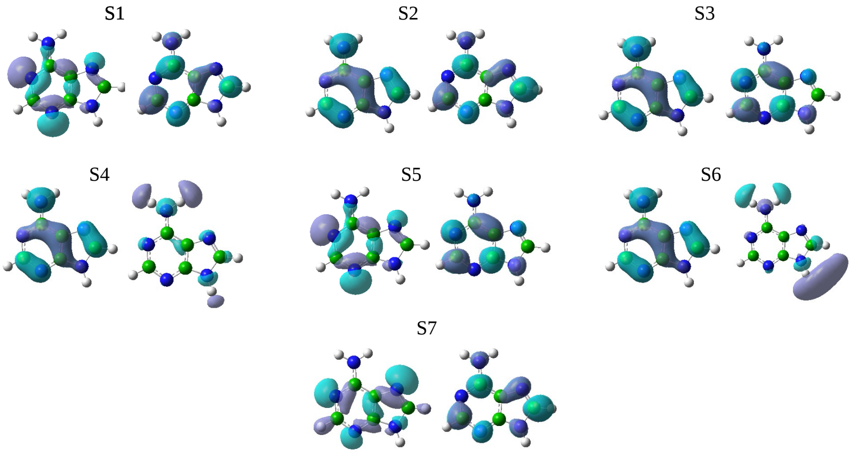

Figure S7: NTOs of Adenine in gas phase at ground state geometry using CAM-B3LYP functional with with an isovalue 0.04 for all orbitals, except the virtual NTOs of the rydberg states which use an isovalue of 0.03.

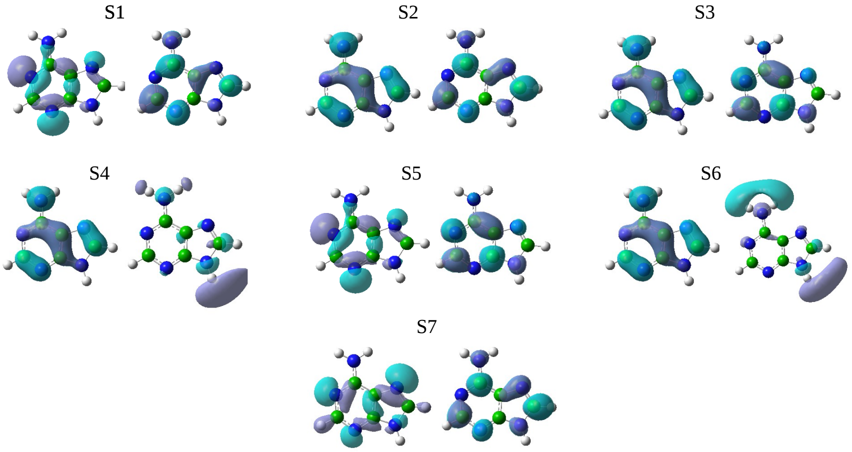

Figure S8: NTOs of Adenine in gas phase at ground state geometry using PBE0 functional with with an isovalue 0.04 for all orbitals, except the virtual NTOs of the rydberg states which use an isovalue of 0.03.

Table S17: Energies of the diabatic states at the FC point and in the minima of each state according to the LVC model for Adenine in C_s symmetry in the gas phase with CAM-B3LYP and PBE0 6-311+G(d,p) calculations.

	$n_N\pi^*1$	L_a	L_b	$\pi Ry_\sigma 1$	$n_N\pi^*2$	$\pi Ry_\sigma 2$	$n_N\pi^*3$
IN MIN ↓	CAM-B3LYP						
S_0	5.369	5.387	5.524	5.865	5.954	6.213	6.305
$n_N\pi^*1$	4.819	5.516	5.999	6.285	6.016	6.626	6.404
L_a	5.275	5.060	5.563	5.809	6.239	6.137	6.362
L_b	5.493	5.299	5.325	5.854	5.861	6.191	6.420
$\pi Ry_\sigma 1$	5.431	5.196	5.505	5.673	6.104	6.000	6.420
$n_N\pi^*2$	5.333	5.797	5.684	6.276	5.502	6.614	6.548
$\pi Ry_\sigma 2$	5.460	5.212	5.531	5.689	6.131	5.985	6.436
$n_N\pi^*3$	5.250	5.451	5.773	6.122	6.078	6.449	5.972
	PBE0						
S_0	5.106	5.160	5.406	5.653	5.732	5.928	6.059
$n_N\pi^*1$	4.585	5.248	5.879	6.031	5.732	6.304	6.087
L_a	4.959	4.874	5.447	5.611	5.915	5.905	6.158
L_b	5.237	5.093	5.228	5.637	5.646	5.917	6.179
$\pi Ry_\sigma 1$	5.174	5.043	5.422	5.442	5.898	5.730	6.223
$n_N\pi^*2$	5.044	5.516	5.600	6.067	5.273	6.338	6.319
$\pi Ry_\sigma 2$	5.170	5.060	5.426	5.453	5.892	5.719	6.194
$n_N\pi^*3$	4.931	5.291	5.666	5.924	5.851	6.172	5.741

Table S18: Energies of the diabatic states at the FC point and in the minima of each state according to the LVC model for Adenine in C_s symmetry in the gas phase with CAM-B3LYP and PBE0 6-31G(d) calculations.

STATE	$n_N\pi^*1$	L_a	L_b	$n_N\pi^*2$	$n_N\pi^*3$
	CAM-B3LYP				
S_0	5.439	5.565	5.636	6.033	6.424
$n_N\pi^*1$	4.860	5.668	6.160	6.109	6.520
L_a	5.292	5.237	5.669	6.252	6.537
L_b	5.584	5.469	5.437	6.033	6.454
$n_N\pi^*2$	5.415	5.934	5.915	5.555	6.679
$n_N\pi^*3$	5.309	5.703	5.820	6.162	6.071
	PBE0				
S_0	5.181	5.315	5.522	5.804	6.169
$n_N\pi^*1$	4.635	5.415	6.017	5.823	6.188
L_a	5.025	5.024	5.562	5.953	6.293
L_b	5.305	5.240	5.347	5.770	6.237
$n_N\pi^*2$	5.131	5.651	5.790	5.327	6.436
$n_N\pi^*3$	4.992	5.486	5.753	5.932	5.831

Table S19: Norm of the coupling vectors for the LVC model in the gas phase at the FC point of Adenine in C_s symmetry obtained by diabatisation at the CAM-B3LYP and PBE0/6-311+G(d,p) level.

STATE	$n_N\pi^*1$	L_a	L_b	$\pi Ry_\sigma 1$	$n_N\pi^*2$	$\pi Ry_\sigma 2$	$n_N\pi^*3$
	CAM-B3LYP						
$n_N\pi^*1$	0.401						
L_a	0.093	0.340					
L_b	0.071	0.126	0.247				
$\pi Ry_\sigma 1$	0.028	0.104	0.103	0.268			
$n_N\pi^*2$	0.237	0.029	0.076	0.054	0.354		
$\pi Ry_\sigma 2$	0.017	0.066	0.055	0.119	0.030	0.296	
$n_N\pi^*3$	0.211	0.139	0.065	0.013	0.078	0.015	0.300
	PBE0						
$n_N\pi^*1$	0.384						
L_a	0.102	0.315					
L_b	0.059	0.126	0.231				
$\pi Ry_\sigma 1$	0.015	0.081	0.097	0.285			
$n_N\pi^*2$	0.229	0.022	0.087	0.032	0.356		
$\pi Ry_\sigma 2$	0.016	0.080	0.065	0.134	0.030	0.292	
$n_N\pi^*3$	0.209	0.129	0.087	0.007	0.052	0.008	0.285

Table S20: Norm of the coupling vectors for the LVC model in the gas phase at the FC point of Adenine in C_s symmetry obtained by diabatisation at the CAM-B3LYP and PBE0/6-31G(d) level.

STATE	$n_N\pi^*1$	L_a	L_b	$n_N\pi^*2$	$n_N\pi^*3$
	CAM-B3LYP				
$n_N\pi^*1$	0.412				
L_a	0.101	0.341			
L_b	0.064	0.138	0.248		
$n_N\pi^*2$	0.243	0.032	0.081	0.364	
$n_N\pi^*3$	0.213	0.116	0.109	0.080	0.308
	PBE0				
$n_N\pi^*1$	0.394				
L_a	0.101	0.318			
L_b	0.060	0.137	0.229		
$n_N\pi^*2$	0.236	0.031	0.084	0.364	
$n_N\pi^*3$	0.212	0.118	0.106	0.052	0.292

S1.5 9H-Guanine

S1.5.1 C_s Symmetry

Table S21: Energies (E_i^0), oscillator strengths f_i, electronic characters and main contributions in terms of transitions among Kohn-Sham orbitals for the lowest excited states of 9H-Guanine used in the LVC model at the ground-state minimum (FC point, C_s symmetry). The predominant character of the states is given, which is the label used on population figs. CAM-B3LYP and PBE0 calculations with 6-311+G(d,p) and 6-31G(d) basis sets in gas phase. Energies in eV.

	CAM-B3LYP				PBE0			
State	E_i^0	f_i	Trans.	Char.	E_i^0	f_i	Trans.	Char.
6-311+G(d,p)								
S_1	5.18	0.1727	H→L+2	L_a	4.86	0.0022	H→L	$\pi Ry_\sigma 1$
S_2	5.22	0.0027	H→L	$\pi Ry_\sigma 1$	5.04	0.1532	H→L+1	L_a
S_3	5.61	0.0002	H-1→L+2	$n_O\pi^*1$	5.36	0.2817	H→L+2	L_b
S_4	5.63	0.3357	H→L+3	L_b	5.39	0.0031	H→L+3	$\pi Ry_\sigma 2$
S_5	5.68	0.0045	H→L+1	$\pi Ry_\sigma 2$	5.47	0.0000	H-1→L+1	$n_O\pi^*1$
S_6	6.30	0.0008	H→L+4	$\pi Ry_\sigma 3$	5.96	0.0012	H→L+4	$\pi Ry_\sigma 3$
S_7	6.36	0.0015	H→L+5	$\pi Ry_\sigma 4$	6.11	0.0005	H-2→L+1	$n_N\pi^*1$
S_8	6.40	0.0028	H-2→L+3	$n_N\pi^*1$	6.15	0.0011	H-2→L+2	$n_N\pi^*2$
S_9	6.49	0.0003	H-2→L+2	$n_N\pi^*2$	6.17	0.0004	H→L+5	$\pi Ry_\sigma 4$
6-31G(d)								
S_1	5.34	0.1628	H→L	L_a	5.18	0.1456	H→L	L_a
S_2	5.63	0.0004	H-1→L	$n_O\pi^*1$	5.49	0.0004	H-1→L	$n_O\pi^*1$
S_3	5.86	0.2919	H→L+1	L_b	5.55	0.2402	H→L+1	L_b
S_4	6.55	0.0037	H-2→L+1	$n_N\pi^*1$	6.20	0.0004	H-2→L	$n_N\pi^*1$
S_5	6.58	0.0011	H-2→L	$n_N\pi^*2$	6.27	0.0019	H-2→L+1	$n_N\pi^*2$

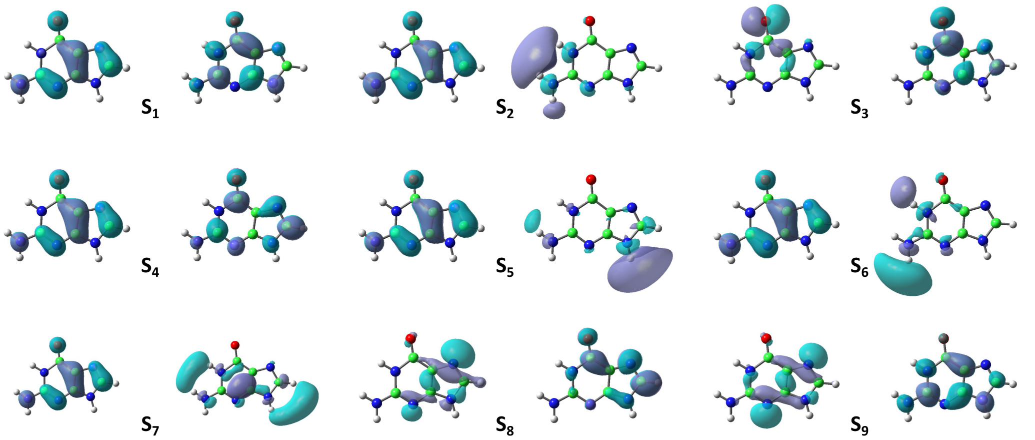

Figure S9: NTOs of 9H-Guanine in gas phase at ground state geometry (C_s symmetry) using the CAM-B3LYP functional and 6-311+G(d,p) basis with an isovalue 0.04 for all orbitals, except the virtual NTOs of the rydberg states which use an isovalue of 0.03.

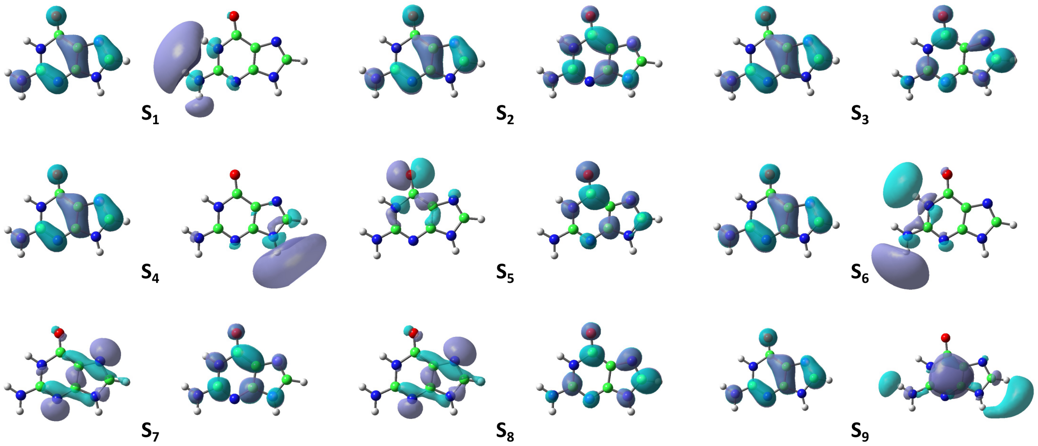

Figure S10: NTOs of 9H-Guanine in gas phase at ground state geometry (C_s symmetry) using the PBE0 functional and 6-311+G(d,p) basis with an isovalue 0.04 for all orbitals, except the virtual NTOs of the rydberg states which use an isovalue of 0.03.

Table S22: Energies of the diabatic states at the FC point and in the minima of each state according to the LVC model for 9H-Guanine in C_s symmetry in the gas phase with CAM-B3LYP and PBE0 6-311+G(d,p) calculations.

	L_a	$\pi Ry_\sigma 1$	$n_O\pi^*1$	L_b	$\pi Ry_\sigma 2$	$\pi Ry_\sigma 3$	$\pi Ry_\sigma 4$	$n_N\pi^*1$	$n_N\pi^*2$
IN MIN ↓	CAM-B3LYP								
S_0	5.185	5.218	5.615	5.634	5.683	6.299	6.360	6.403	6.485
L_a	4.843	5.208	5.792	5.557	5.624	6.294	6.332	6.519	6.272
$\pi Ry_\sigma 1$	5.131	4.920	5.996	5.443	5.444	6.065	6.100	6.609	6.555
$n_O\pi^*1$	5.464	5.745	5.171	5.967	6.162	6.839	6.861	6.576	6.818
L_b	5.129	5.092	5.866	5.272	5.528	6.196	6.221	6.412	6.575
$\pi Ry_\sigma 2$	5.103	5.000	5.968	5.435	5.365	6.116	6.091	6.583	6.559
$\pi Ry_\sigma 3$	5.098	4.946	5.971	5.429	5.442	6.039	6.115	6.563	6.520
$\pi Ry_\sigma 4$	5.111	4.956	5.967	5.427	5.391	6.089	6.065	6.575	6.553
$n_N\pi^*1$	5.544	5.711	5.928	5.865	6.129	6.783	6.821	5.819	6.483
$n_N\pi^*2$	5.227	5.588	6.101	5.959	6.036	6.671	6.730	6.414	5.888
	PBE0								
S_0	5.039	4.861	5.466	5.359	5.390	5.964	6.173	6.112	6.148
L_a	4.741	4.826	5.551	5.298	5.320	5.926	6.132	5.939	6.190
$\pi Ry_\sigma 1$	5.048	4.519	5.883	5.255	5.224	5.653	6.000	6.301	6.262
$n_O\pi^*1$	5.298	5.409	4.993	5.710	5.878	6.542	6.690	6.303	6.356
L_b	4.981	4.716	5.645	5.058	5.271	5.807	6.064	6.239	6.023
$\pi Ry_\sigma 2$	4.978	4.661	5.788	5.246	5.083	5.756	5.978	6.243	6.260
$\pi Ry_\sigma 3$	5.049	4.553	5.917	5.246	5.220	5.619	6.004	6.309	6.261
$\pi Ry_\sigma 4$	4.940	4.585	5.750	5.188	5.127	5.689	5.934	6.170	6.160
$n_N\pi^*1$	5.033	5.173	5.650	5.651	5.679	6.281	6.457	5.647	5.938
$n_N\pi^*2$	5.254	5.104	5.672	5.403	5.666	6.203	6.417	5.908	5.677

Table S23: Energies of the diabatic states at the FC point and in the minima of each state according to the LVC model for 9H-Guanine in C_s symmetry in the gas phase with CAM-B3LYP and PBE0 6-31G(d) calculations.

	L_a	$n_O\pi^*1$	L_b	$n_N\pi^*1$	$n_N\pi^*2$
IN MIN ↓	CAM-B3LYP				
S_0	5.338	5.630	5.860	6.553	6.582
L_a	4.993	5.810	5.780	6.681	6.336
$n_O\pi^*1$	5.661	5.142	6.210	6.818	6.919
L_b	5.285	5.863	5.488	6.485	6.735
$n_N\pi^*1$	5.722	6.007	6.021	5.952	6.530
$n_N\pi^*2$	5.348	6.080	6.242	6.502	5.981
	PBE0				
S_0	5.185	5.491	5.549	6.204	6.274
L_a	4.884	5.578	5.499	5.990	6.324
$n_O\pi^*1$	5.479	4.983	5.917	6.483	6.421
L_b	5.134	5.652	5.248	6.210	6.260
$n_N\pi^*1$	5.210	5.802	5.794	5.664	6.201
$n_N\pi^*2$	5.442	5.638	5.743	6.099	5.766

Table S24: Norm of the coupling vectors for the LVC model in the gas phase at the FC point of 9H-Guanine with C_s symmetry obtained by diabatisation at the CAM-B3LYP and PBE0/6-311+G(d,p) level.

STATE	L_a	$\pi Ry_\sigma 1$	$n_O\pi^*1$	L_b	$\pi Ry_\sigma 2$	$\pi Ry_\sigma 3$	$\pi Ry_\sigma 4$	$n_N\pi^*1$	$n_N\pi^*2$
	CAM-B3LYP								
L_a	0.333								
$\pi Ry_\sigma 1$	0.112	0.339							
$n_O\pi^*1$	0.049	0.012	0.439						
L_b	0.159	0.089	0.038	0.359					
$\pi Ry_\sigma 2$	0.096	0.085	0.061	0.064	0.340				
$\pi Ry_\sigma 3$	0.098	0.125	0.010	0.091	0.076	0.318			
$\pi Ry_\sigma 4$	0.161	0.097	0.020	0.088	0.114	0.076	0.324		
$n_N\pi^*1$	0.048	0.008	0.107	0.124	0.014	0.028	0.018	0.421	
$n_N\pi^*2$	0.140	0.009	0.061	0.055	0.010	0.012	0.028	0.171	0.395
	PBE0								
L_a	0.309								
$\pi Ry_\sigma 1$	0.104	0.377							
$n_O\pi^*1$	0.041	0.012	0.455						
L_b	0.160	0.076	0.038	0.325					
$\pi Ry_\sigma 2$	0.091	0.071	0.035	0.053	0.343				
$\pi Ry_\sigma 3$	0.108	0.162	0.008	0.052	0.060	0.380			
$\pi Ry_\sigma 4$	0.154	0.088	0.023	0.118	0.102	0.058	0.291		
$n_N\pi^*1$	0.142	0.013	0.107	0.032	0.017	0.012	0.058	0.363	
$n_N\pi^*2$	0.042	0.013	0.134	0.123	0.017	0.018	0.055	0.219	0.368

Table S25: Norm of the coupling vectors for the LVC model in the gas phase at the FC point of 9H-Guanine with C_s symmetry obtained by diabatisation at the CAM-B3LYP and PBE0/6-31G(d) level.

STATE	L_a	$n_O\pi^*1$	L_b	$n_N\pi^*1$	$n_N\pi^*2$
	CAM-B3LYP				
L_a	0.337				
$n_O\pi^*1$	0.039	0.466			
L_b	0.179	0.039	0.366		
$n_N\pi^*1$	0.026	0.096	0.134	0.422	
$n_N\pi^*2$	0.150	0.075	0.031	0.194	0.404
	PBE0				
L_a	0.312				
$n_O\pi^*1$	0.041	0.477			
L_b	0.180	0.040	0.326		
$n_N\pi^*1$	0.126	0.106	0.046	0.397	
$n_N\pi^*2$	0.063	0.146	0.121	0.207	0.385

S1.5.2 No Symmetry

Table S26: Energies (E_i^0), oscillator strengths f_i, electronic characters and main contributions in terms of transitions among Kohn-Sham orbitals for the lowest excited states of 9H-Guanine used in the LVC model at the ground-state minimum (FC point, no symmetry). The predominant character of the states is given, which is the label used on population figs. When the states are mixed, smaller contributions are indicated in parentheses. CAM-B3LYP and PBE0 calculations with 6-311+G(d,p) and 6-31G(d) basis sets in gas phase. PBE0/6-31G(d) includes $n_O\pi^*2$ as it is closer in energy to other states than at other levels of theory and the $n\pi^*$ states are quite mixed. Energies in eV.

	CAM-B3LYP				PBE0			
State	E_i^0	f_i	Trans.	Char.	E_i^0	f_i	Trans.	Char.
				6-311+G(d,p)				
S_1	5.17	0.1723	H→L+2	L_a	4.95	0.0783	H→L	$\pi Ry_\sigma 1$ (+ $\pi\pi^*$)
S_2	5.33	0.0089	H→L	$\pi Ry_\sigma 1$	5.07	0.0896	H→L+1	L_a (+ πRy_σ)
S_3	5.60	0.0034	H-1→L+3	$n_O\pi^*1$	5.36	0.2513	H→L+2	L_b
S_4	5.65	0.3087	H→L+3	L_b	5.44	0.0032	H-1→L+2<br>H-1→L+1	$n_O\pi^*1$
S_5	5.77	0.0113	H→L+1	$\pi Ry_\sigma 2$	5.48	0.0073	H→L+3	$\pi Ry_\sigma 2$
S_6	6.39	0.0019	H-2→L+3	$n_N\pi^*1$	6.02	0.0002	H-1→L<br>H-2→L	$n_N\pi^*1$ (+ $n_O\pi^*$)
S_7	6.41	0.0011	H→L+4	$\pi Ry_\sigma 3$	6.10	0.0023	H→L+4	$\pi Ry_\sigma 3$
S_8	6.42	0.0019	H-2→L+2	$n_N\pi^*2$	6.14	0.0013	H-2→L+2	$n_N\pi^*2$
				6-31G(d)				
S_1	5.30	0.1774	H→L	L_a	5.13	0.1689	H→L	L_a
S_2	5.60	0.0012	H-1→L+1	$n_O\pi^*1$	5.47	0.0033	H-1→L+1	$n_O\pi^*1$
S_3	5.87	0.2646	H→L+1	L_b	5.57	0.2055	H→L+1	L_b
S_4	6.46	0.0002	H-2→L	$n_N\pi^*1$	6.06	0.0004	H-1→L	$n_N\pi^*1$ (+$n_O\pi^*$)
S_5	6.56	0.0050	H-2→L+1	$n_N\pi^*2$	6.26	0.0013	H-2→L<br>H-2→L+1	$n_N\pi^*2$ (+$n_O\pi^*$)
S_6					6.33	0.0011	H-2→L+1	$n_O\pi^*2$ (+$n_N\pi^*$)

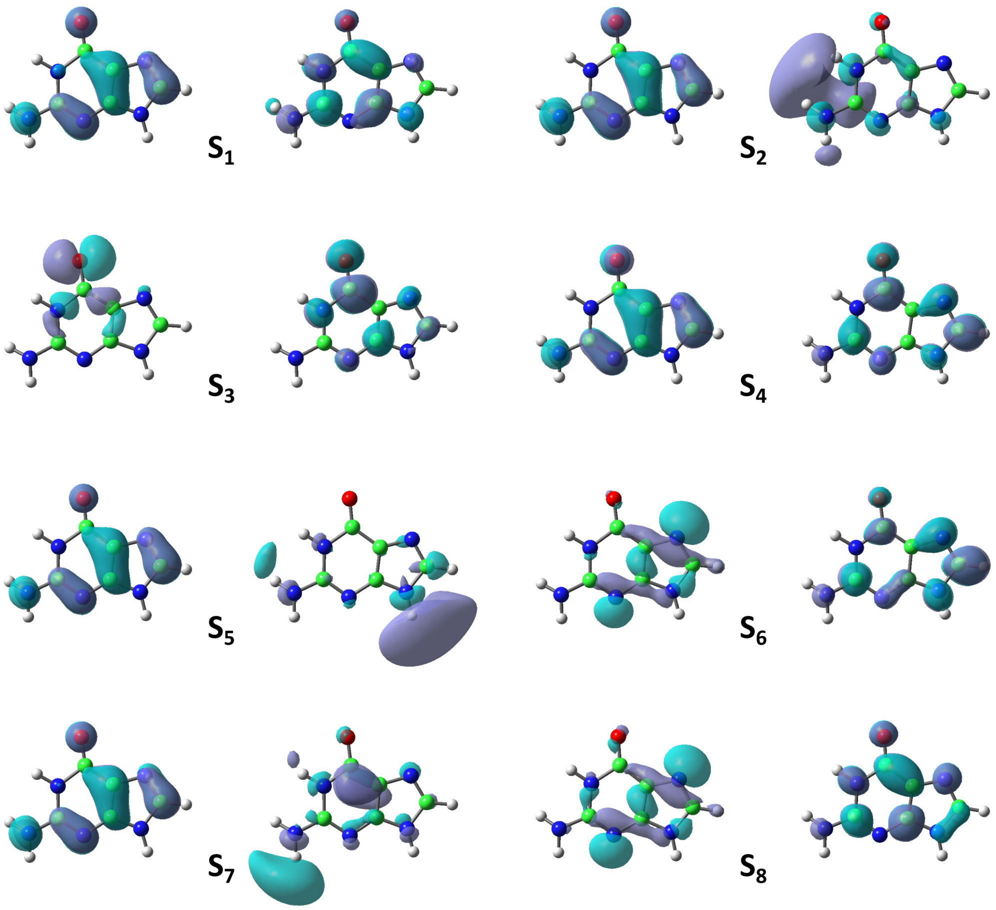

Figure S11: NTOs of 9H-Guanine in gas phase at ground state geometry (no symmetry) using the CAM-B3LYP functional and 6-311+G(d,p) basis with an isovalue 0.04 for all orbitals, except the virtual NTOs of the rydberg states which use an isovalue of 0.03.

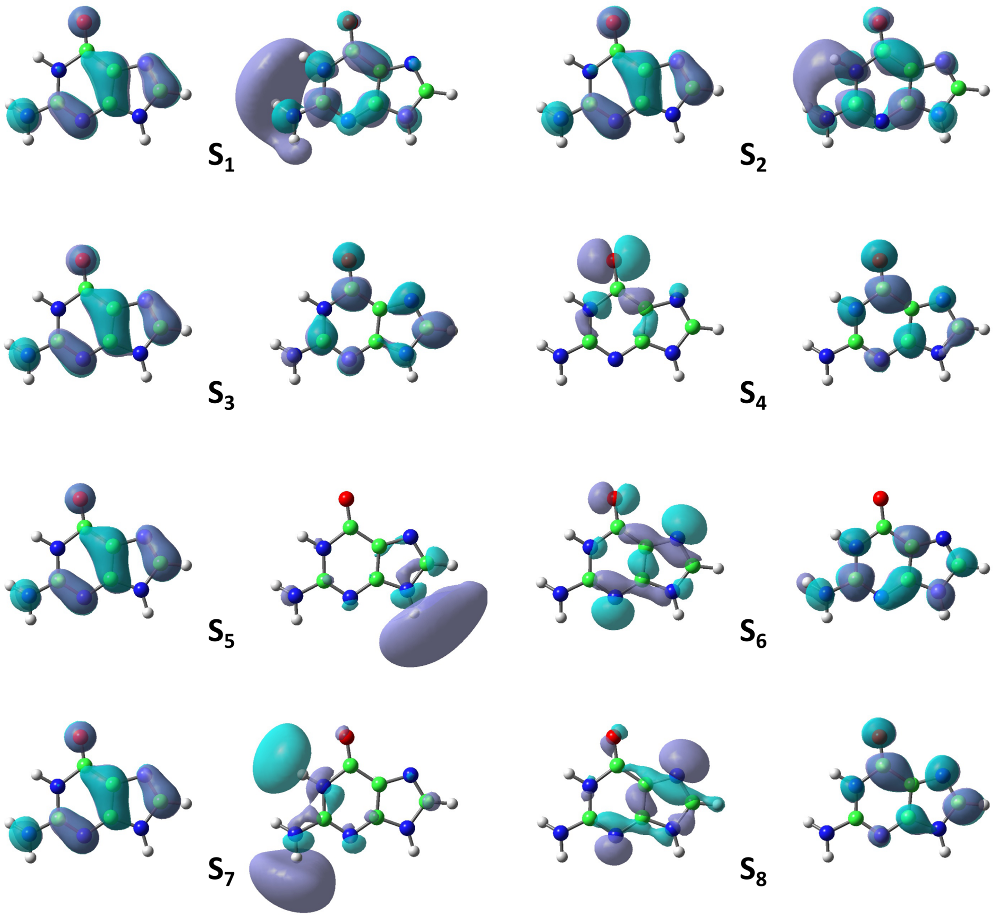

Figure S12: NTOs of 9H-Guanine in gas phase at ground state geometry (no symmetry) using the PBE0 functional and 6-311+G(d,p) basis with an isovalue 0.04 for all orbitals, except the virtual NTOs of the rydberg states which use an isovalue of 0.03.

Table S27: Energies of the diabatic states of LVC model on all minima of 9H-Guanine without symmetry restrictions in gas phase with CAM-B3LYP and PBE0/6-311+G(d,p) calculation.

	L_a	$\pi Ry_\sigma 1$	$n_O\pi^*1$	L_b	$\pi Ry_\sigma 2$	$n_N\pi^*1$	$\pi Ry_\sigma 3$	$n_N\pi^*2$
IN MIN ↓	CAM-B3LYP							
S_0	5.171	5.330	5.598	5.655	5.774	6.390	6.408	6.422
L_a	4.814	5.295	5.809	5.580	5.701	6.447	6.377	6.307
$\pi Ry_\sigma 1$	5.252	4.857	6.198	5.551	5.396	6.628	6.152	6.846
$n_O\pi^*1$	5.477	5.908	5.147	5.989	6.316	6.738	6.958	6.607
L_b	5.102	5.116	5.843	5.292	5.540	6.309	6.253	6.633
$\pi Ry_\sigma 2$	5.204	4.942	6.152	5.522	5.310	6.609	6.160	6.787
$n_N\pi^*1$	5.292	5.516	5.915	5.633	5.950	5.969	6.500	6.226
$\pi Ry_\sigma 3$	5.109	4.927	6.023	5.463	5.388	6.387	6.082	6.591
$n_N\pi^*2$	5.200	5.781	5.833	6.004	6.177	6.274	6.752	5.921
	PBE0							
S_0	5.068	4.947	5.445	5.381	5.479	6.020	6.104	6.139
L_a	4.692	4.882	5.719	5.320	5.303	6.101	5.821	6.325
$\pi Ry_\sigma 1$	5.034	4.540	5.856	5.341	5.293	6.120	5.762	6.418
$n_O\pi^*1$	5.422	5.407	4.989	5.707	5.955	6.239	6.691	6.222
L_b	4.927	4.796	5.611	5.085	5.300	6.084	5.862	6.167
$\pi Ry_\sigma 2$	4.905	4.743	5.854	5.295	5.090	6.320	5.716	6.411
$n_N\pi^*1$	5.409	5.277	5.844	5.785	6.026	5.383	6.701	6.221
$\pi Ry_\sigma 3$	4.952	4.741	6.120	5.386	5.245	6.524	5.560	6.590
$n_N\pi^*2$	5.350	5.290	5.544	5.584	5.833	5.937	6.483	5.667

Table S28: Energies of the diabatic states of LVC model on all minima of 9H-Guanine without symmetry restrictions in gas phase with CAM-B3LYP and PBE0/6-31G(d) calculation. Only PBE0 LVC potential includes the $n_O\pi^*2$ state, as it is found close in energy to the other $n\pi^*$ states.

	L_a	$n_O\pi^*1$	L_b	$n_N\pi^*1$	$n_N\pi^*2$	$n_O\pi^*2$
IN MIN ↓	CAM-B3LYP					
S_0	5.303	5.604	5.873	6.457	6.564	-
L_a	4.915	5.822	5.834	6.207	6.705	-
$n_O\pi^*1$	5.629	5.108	6.227	6.842	6.754	-
L_b	5.229	5.814	5.521	6.511	6.614	-
$n_N\pi^*1$	5.406	6.234	6.316	5.716	6.725	-
$n_N\pi^*2$	5.661	5.903	6.175	6.482	5.959	-
	PBE0					
S_0	5.132	5.465	5.571	6.057	6.256	6.331
L_a	4.759	5.601	5.623	5.713	6.216	6.229
$n_O\pi^*1$	5.406	4.953	5.933	6.258	6.259	6.352
L_b	5.099	5.603	5.283	6.145	6.402	6.402
$n_N\pi^*1$	5.149	5.889	6.105	5.323	6.288	6.053
$n_N\pi^*2$	5.122	5.360	5.832	5.758	5.853	6.104
$n_O\pi^*2$	5.129	5.447	5.826	5.517	6.098	5.859

Table S29: Norm of the coupling vectors for the LVC model in the gas phase at the FC point of 9H-Guanine without symmetry restrictions obtained by diabatisation at the CAM-B3LYP and PBE0/6-311+G(d,p) level.

STATE	$\pi\pi^*1$	$\pi Ry_\sigma 1$	$n_O\pi^*1$	$\pi\pi^*2$	$\pi Ry_\sigma 2$	$n_N\pi^*1$	$\pi Ry_\sigma 3$	$n_N\pi^*2$
	CAM-B3LYP							
$\pi\pi^*1$	0.345							
$\pi Ry_\sigma 1$	0.146	0.344						
$n_O\pi^*1$	0.046	0.024	0.440					
$\pi\pi^*2$	0.163	0.100	0.068	0.353				
$\pi Ry_\sigma 2$	0.091	0.095	0.026	0.069	0.362			
$n_N\pi^*1$	0.072	0.044	0.066	0.131	0.034	0.347		
$\pi Ry_\sigma 3$	0.110	0.097	0.025	0.089	0.083	0.147	0.301	
$n_N\pi^*2$	0.138	0.037	0.102	0.052	0.017	0.205	0.088	0.384
	PBE0							
$\pi\pi^*1$	0.316							
$\pi Ry_\sigma 1$	0.170	0.364						
$n_O\pi^*1$	0.043	0.031	0.445					
$\pi\pi^*2$	0.144	0.115	0.061	0.316				
$\pi Ry_\sigma 2$	0.078	0.083	0.083	0.061	0.350			
$n_N\pi^*1$	0.085	0.091	0.103	0.050	0.019	0.409		
$\pi Ry_\sigma 3$	0.115	0.148	0.021	0.056	0.068	0.039	0.383	
$n_N\pi^*2$	0.064	0.044	0.146	0.114	0.026	0.182	0.078	0.375

Table S30: Norm of the coupling vectors for the LVC model in the gas phase at the FC point of 9H-Guanine without symmetry restrictions obtained by diabatisation at the CAM-B3LYP and PBE0/6-31G(d) level. Only PBE0 LVC potential includes the $n_O\pi^*2$ state, as it is found close in energy to the other $n\pi^*$ states.

STATE	L_a	$n_O\pi^*1$	L_b	$n_N\pi^*1$	$n_N\pi^*2$	$n_O\pi^*2$
	CAM-B3LYP					
L_a	0.360					
$n_O\pi^*1$	0.041	0.466				
L_b	0.192	0.046	0.352			
$n_N\pi^*1$	0.134	0.051	0.072	0.422		
$n_N\pi^*2$	0.071	0.111	0.116	0.181	0.432	
	PBE0					
L_a	0.348					
$n_O\pi^*1$	0.046	0.475				
L_b	0.185	0.068	0.310			
$n_N\pi^*1$	0.117	0.121	0.052	0.441		
$n_N\pi^*2$	0.101	0.165	0.090	0.199	0.361	
$n_O\pi^*2$	0.070	0.110	0.110	0.179	0.279	0.373

S1.6 7H-Guanine

Table S31: Energies (E_i^0), oscillator strengths f_i, electronic characters and main contributions in terms of transitions among Kohn-Sham orbitals for the lowest excited states of 7H-Guanine used in the LVC model at the ground-state minimum (FC point, C_s symmetry). The predominant character of the states is given, which is the label used on population figs. CAM-B3LYP and PBE0 calculations with 6-311+G(d,p) basis set in gas phase. Energies in eV.

	CAM-B3LYP				PBE0			
State	E_i^0	f_i	Trans.	Char.	E_i^0	f_i	Trans.	Char.
S_1	4.91	0.1508	H→L+1	L_a	4.72	0.1274	H→L	L_a
S_2	5.30	0.0036	H→L	$\pi Ry_\sigma 1$	4.98	0.0027	H→L+1	$\pi Ry_\sigma 1$
S_3	5.51	0.0001	H-1→L+1	$n_O\pi^*1$	5.25	0.0001	H-1→L	$n_O\pi^*1$
				$(+n_{N9}\pi^*)$				$(+n_{N9}\pi^*)$
S_4	5.73	0.0051	H→L+2	$\pi Ry_\sigma 2$	5.47	0.0038	H→L+2	$\pi Ry_\sigma 2$
S_5	5.87	0.1575	H→L+3	L_b	5.59	0.1218	H→L+3	L_b
S_6	6.07	0.0003	H-3→L+1	$n_{N9+N3}\pi^*1$	5.76	0.0000	H-3→L	$n_{N9+N3}\pi^*1$
				$(+n_O\pi^*)$				$(+n_O\pi^*)$

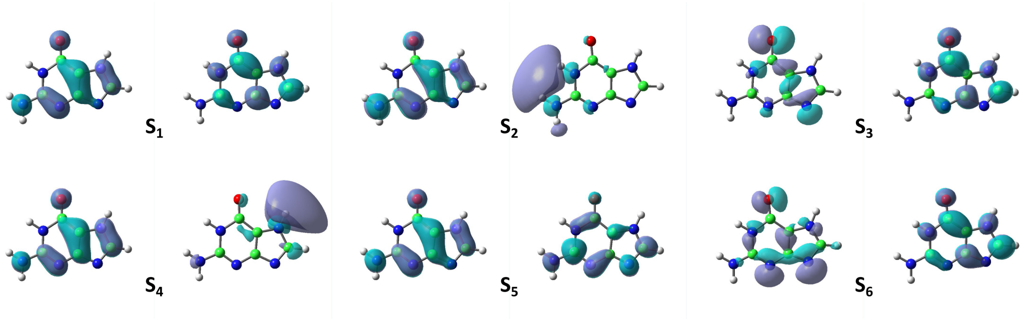

Figure S13: NTOs of 7H-Guanine in gas phase at ground state geometry (C_s symmetry) using the CAM-B3LYP functional and 6-311+G(d,p) basis with an isovalue 0.04 for all orbitals, except the virtual NTOs of the rydberg states which use an isovalue of 0.03.

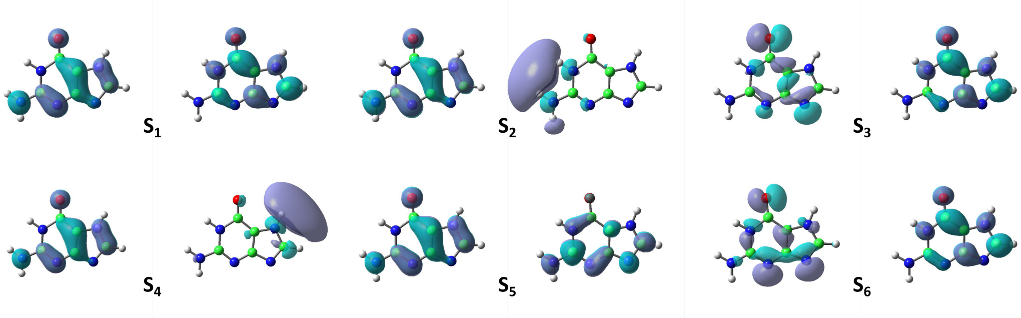

Figure S14: NTOs of 7H-Guanine in gas phase at ground state geometry (C_s symmetry) using the PBE0 functional and 6-311+G(d,p) basis with an isovalue 0.04 for all orbitals, except the virtual NTOs of the rydberg states which use an isovalue of 0.03.

Table S32: Energies of the diabatic states at the FC point and in the minima of each state according to the LVC model for 7H-Guanine in C_s symmetry in the gas phase with CAM-B3LYP and PBE0 6-311+G(d,p) calculations.

	L_a	$\pi Ry_\sigma 1$	$n_O\pi^*1$ (+$n_{N9}\pi^*$)	$\pi Ry_\sigma 2$	L_b	$n_{N9+N3}\pi^*1$ (+$n_O\pi^*$)
IN MIN ↓			CAM-B3LYP			
S_0	4.907	5.296	5.511	5.727	5.866	6.070
L_a	4.594	5.308	5.561	5.687	5.757	6.026
$\pi Ry_\sigma 1$	4.895	5.007	5.881	5.569	5.666	6.451
$n_O\pi^*1$ (+$n_{N9}\pi^*$)	4.999	5.732	5.156	6.169	6.164	5.885
$\pi Ry_\sigma 2$	4.815	5.109	5.858	5.466	5.696	6.367
L_b	4.865	5.188	5.835	5.677	5.485	6.409
$n_{N9+N3}\pi^*1$ (+$n_O\pi^*$)	4.920	5.759	5.342	6.134	6.195	5.699
			PBE0			
S_0	4.719	4.979	5.250	5.471	5.590	5.765
L_a	4.466	4.994	5.237	5.469	5.518	5.686
$\pi Ry_\sigma 1$	4.816	4.644	5.650	5.395	5.461	6.236
$n_O\pi^*1$ (+$n_{N9}\pi^*$)	4.832	5.423	4.872	5.943	5.865	5.551
$\pi Ry_\sigma 2$	4.710	4.813	5.588	5.226	5.483	6.080
L_b	4.747	4.868	5.499	5.471	5.237	6.106
$n_{N9+N3}\pi^*1$ (+$n_O\pi^*$)	4.773	5.500	5.043	5.926	5.964	5.380

Table S33: Norm of the coupling vectors for the LVC model in the gas phase at the FC point of 7H-Guanine with C_s symmetry obtained by diabatisation at the CAM-B3LYP and PBE0/6-311+G(d,p) level.

STATE	L_a	$\pi Ry_\sigma 1$	$n_O\pi^*1$ $(+n_{N9}\pi^*)$	$\pi Ry_\sigma 2$	L_b	$n_{N9+N3}\pi^*1$ $(+n_O\pi^*)$
	CAM-B3LYP					
L_a	0.316					
$\pi Ry_\sigma 1$	0.078	0.337				
$n_O\pi^*1$ $(+n_{N9}\pi^*)$	0.065	0.016	0.371			
$\pi Ry_\sigma 2$	0.086	0.084	0.031	0.318		
L_b	0.160	0.112	0.035	0.079	0.364	
$n_{N9+N3}\pi^*1$ $(+n_O\pi^*)$	0.108	0.007	0.222	0.017	0.043	0.346
	PBE0					
L_a	0.278					
$\pi Ry_\sigma 1$	0.069	0.374				
$n_O\pi^*1$ $(+n_{N9}\pi^*)$	0.067	0.018	0.376			
$\pi Ry_\sigma 2$	0.071	0.065	0.026	0.320		
L_b	0.159	0.105	0.035	0.068	0.347	
$n_{N9+N3}\pi^*1$ $(+n_O\pi^*)$	0.103	0.008	0.224	0.014	0.042	0.357

S2 Additional Dynamics and Spectra

In this section are additional dynamics calculations with a 6-31G(d) basis set. Most of the results are similar to the 6-311+G(d,p) results presented in the main text, except when Rydberg states are involved in the dynamics, as these are not included when using a 6-31G(d) basis set.

Also presented are calculated spectra from the LVC model with and without inter-state coupling, showing the absolute calculated intensities and contributions from individual states.

S2.1 Uracil

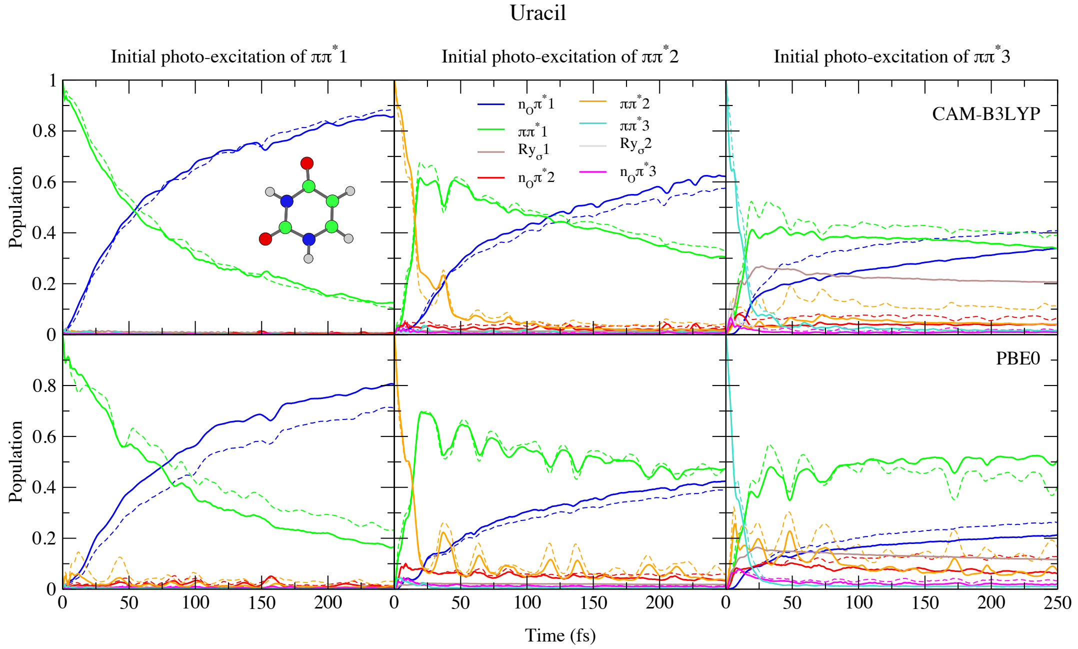

Figure S15: Nonadiabatic dynamics of electronic populations of Uracil in the gas phase, as predicted by an LVC Hamiltonian parameterized with calculations at the FC point using CAM-B3LYP (top) and PBE0 (bottom) functionals with 6-311+G(d,p) (solid lines) and 6-31G(d) (dashed lines).

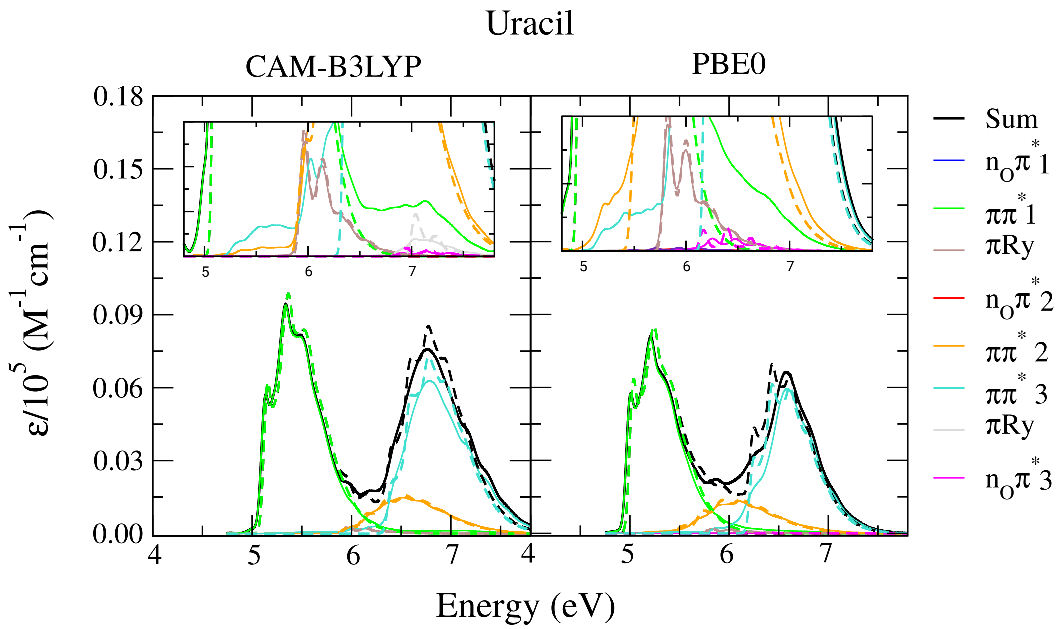

Figure S16: Absorption spectra of Uracil including individual state components from LVC models parametrized by CAM-B3LYP (left) and PBE0 (right) calculations, with 6-311+G(d,p) basis set and with (solid) and without (dashed) inter-state couplings.

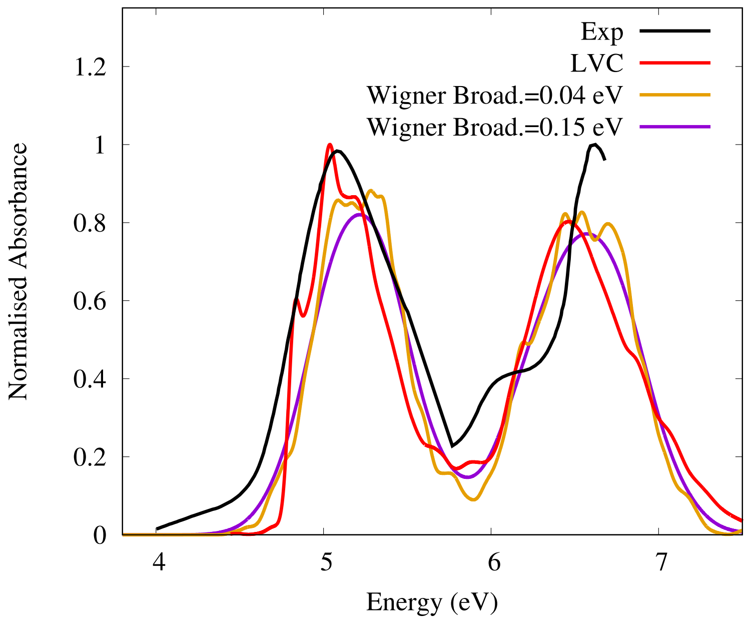

Figure S17: Absorption spectra of Uracil from experiment, and calculated at 0 K via the LVC model, and from sampling from the Wigner distribution. The Wigner distribution includes two different Gaussian broadening values (HWHM=0.04 and 0.15 eV) whereas the LVC spectrum is only computed with a broadeningHWHM=0.04 eV. LVC parametrized and Wigner calculated using CAM-B3LYP/6-311+G(d,p) computations.

In Fig. S17 is shown absorption spectra of Ura calculated classically via sampling from the Wigner distribution (neglecting inter-state couplings), compared to the quantum vibronic LVC spectrum and experimental spectrum shown in the main text. The Wigner spectra are shown with two different broadening values, 0.15 eV

and 0.04 eV. By construction, for each final state the Wigner spectrum reproduces the first and second moment of the quantum LVC spectrum (with the inter-state couplings set to zero). However it does not reproduce higher moments (the asymmetry of the shape) and cannot describe individual vibronic bands.[1] Therefore the intensity modulation in the 0.04 eV broadened spectrum does not reflect individual vibronic bands, but is simply due to an insufficient sampling (although 20000 points were used, notice that it has been shown that using machine learning the convergence could be accelerated[2]). Apart from that, the two bands of the Wigner spectra appear more symmetric while in the LVC spectrum they are more asymmetric with a sharper onset at low-energies and a longer high-energy tail. As a consequence, since the first (M_1) and second (M_2) moments are the same (apart from the effect of the inter-state couplings on the M_2), the Wigner spectrum shows a more gradual onset on the red-wing, which should be considered spurious. Notice in fact that this latter is not due either to temperature (set to 0 K) or to any intensity borrowing of the nπ^* state (inter-state couplings are set to zero). We also increased the phenomenological broadening to 0.15 eV so to have a Wigner spectrum in which the intensity modulation is washed out. In this case clearly also the red-wing becomes longer improving the agreement with experiment. However, this result should be considered as arising from a compensation of errors, on one side the classical approximation of the spectrum, on the other side the limitations of the harmonic approximation and the temperature set to 0 (while the experimental spectrum was recorded at 501 K). Additionally the Wigner spectrum does not reproduce the vibronic shoulder at $\sim$4.8 eV. Finally, since in the reported calculations the Wigner approach doesn't account for the inter-state coupling, we can conclude that the higher relative intensity between the two bands (at $\sim$ 6 eV) and the smoothness of the high energy peak for the 0.15 eV broadened spectrum are simply a function of this broadening value, rather than the physical effect of ultrafast internal conversion, captured in the LVC spectrum.

S2.2 Thymine

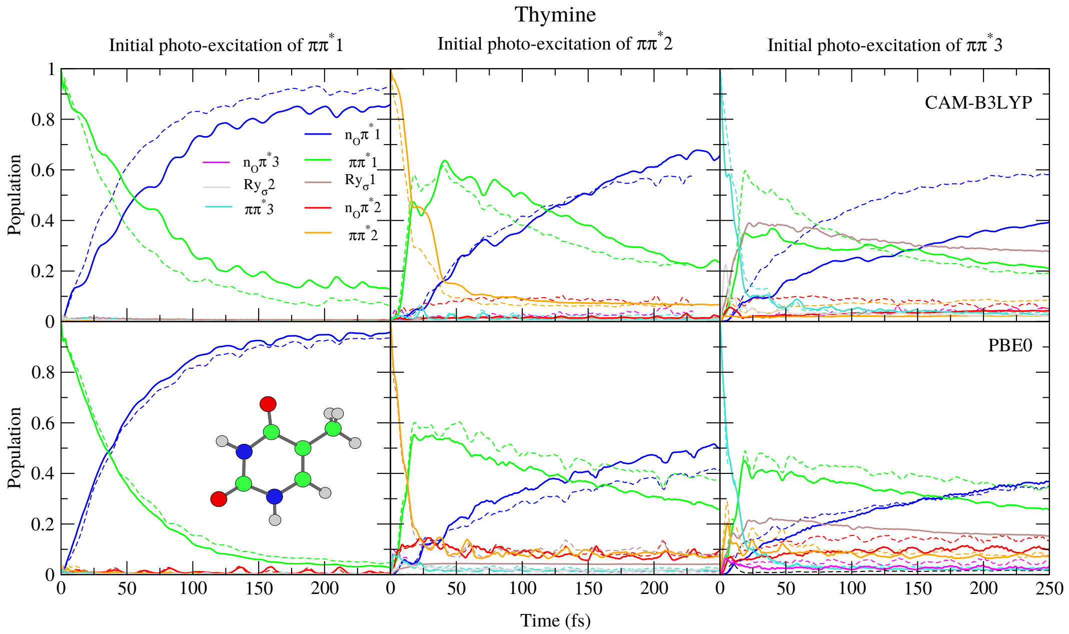

Figure S18: Nonadiabatic dynamics of electronic populations of Thymine in the gas phase, as predicted by an LVC Hamiltonian parameterized with calculations at the FC point using CAM-B3LYP (top) and PBE0 (bottom) functionals with 6-311+G(d,p) (solid lines) and 6-31G(d) (dashed lines).

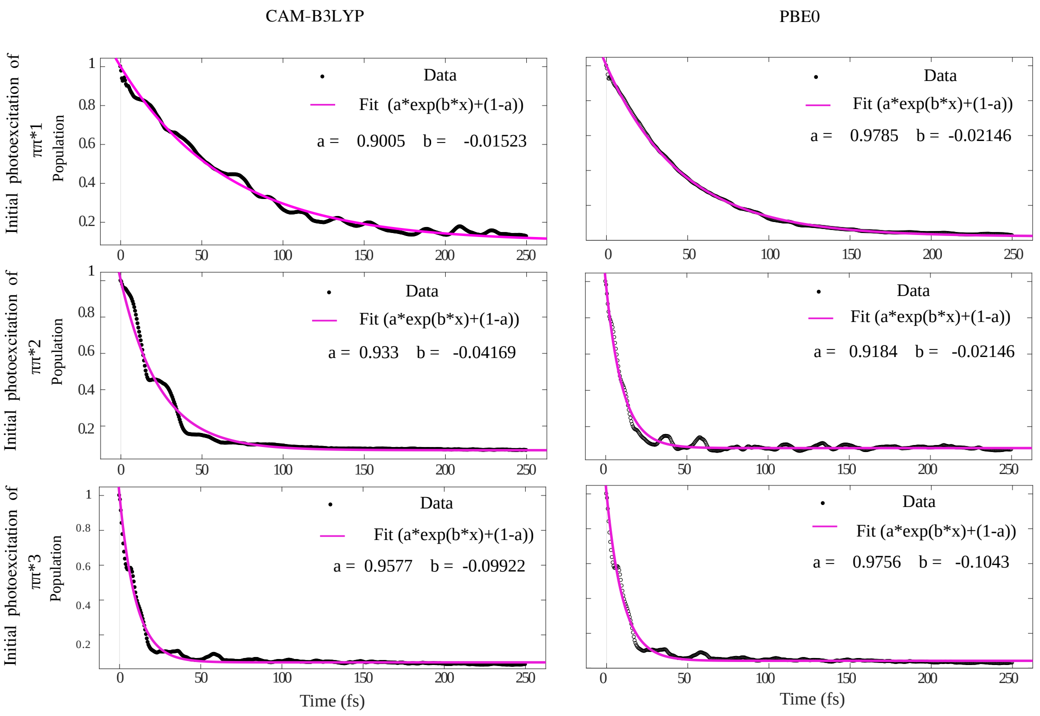

Figure S19: Mono-exponential fit of the decay of bright states for Thymine with initial photoexcitation of $\pi\pi^*1$(top), $\pi\pi^*2$(middle) and $\pi\pi^*3$(bottom) using CAM-B3LYP (left) and PBE0 (right) functionals.

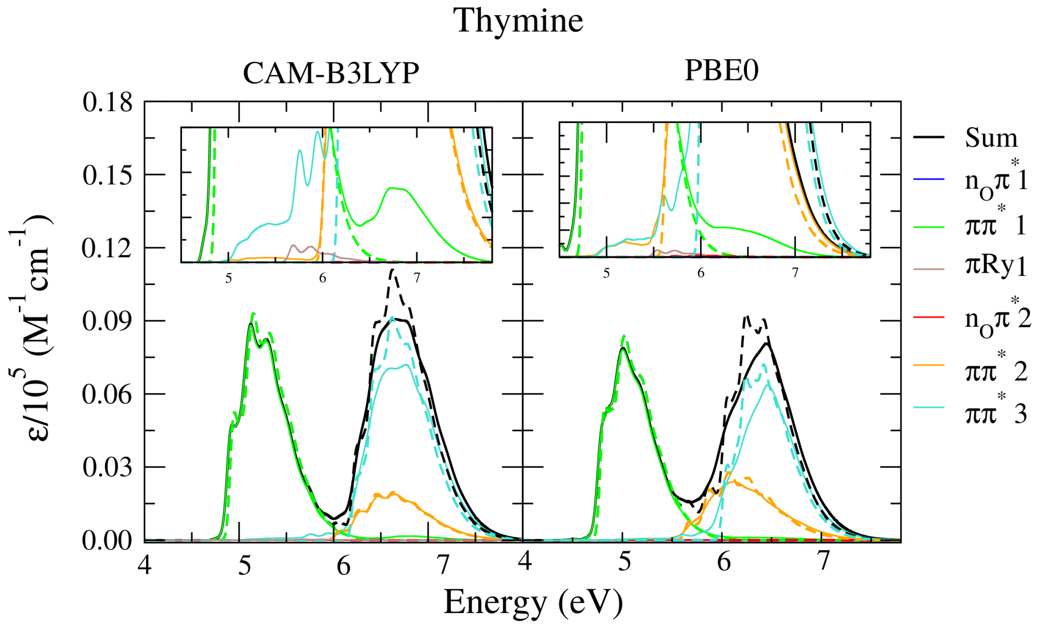

Figure S20: Absorption spectra of Thymine including individual state components from LVC models parametrized by CAM-B3LYP (left) and PBE0 (right) calculations, with 6-311+G(d,p) basis set and with (solid) and without (dashed) inter-state couplings.

S2.3 Cytosine

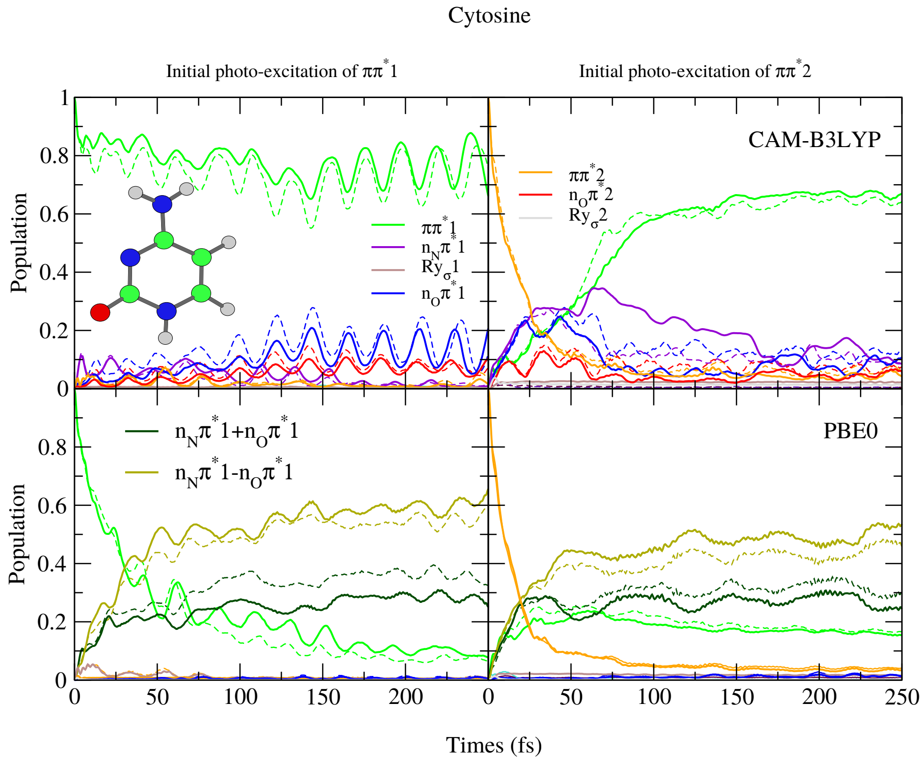

Figure S21: Nonadiabatic dynamics of electronic populations of Cytosine in the gas phase, as predicted by an LVC Hamiltonian parameterized with calculations at the FC point using CAM-B3LYP (top) and PBE0 (bottom) functionals with 6-311+G(d,p) (solid lines) and 6-31G(d) (dashed lines).

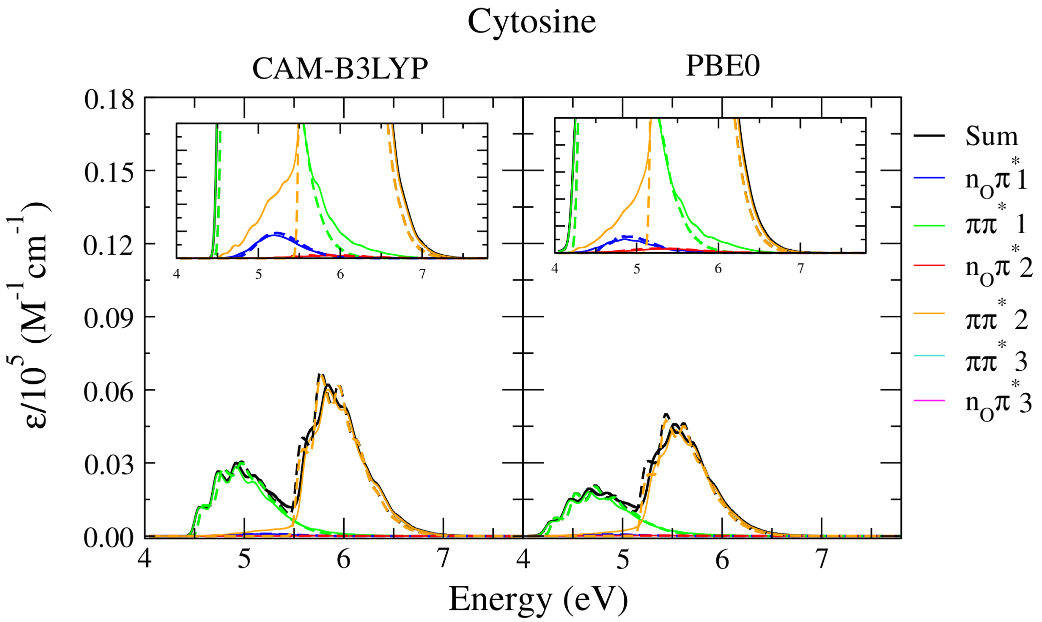

Figure S22: Absorption spectra of Cytosine including individual state components from LVC models parametrized by CAM-B3LYP (left) and PBE0 (right) calculations, with 6-311+G(d,p) basis set and with (solid) and without (dashed) inter-state couplings.

S2.4 Adenine

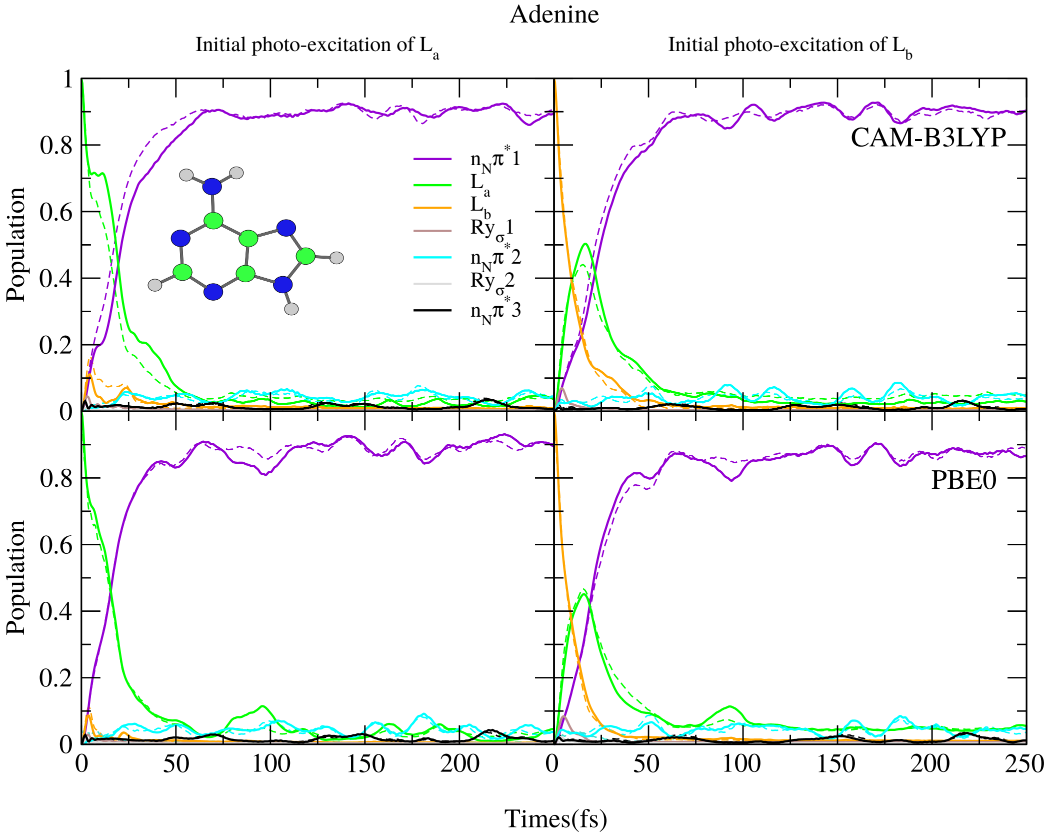

Figure S23: Nonadiabatic dynamics of electronic populations of Adenine in the gas phase, as predicted by an LVC Hamiltonian parameterized with calculations at the FC point using CAM-B3LYP (top) and PBE0 (bottom) functionals with 6-311+G(d,p) (solid lines) and 6-31G(d) (dashed lines).

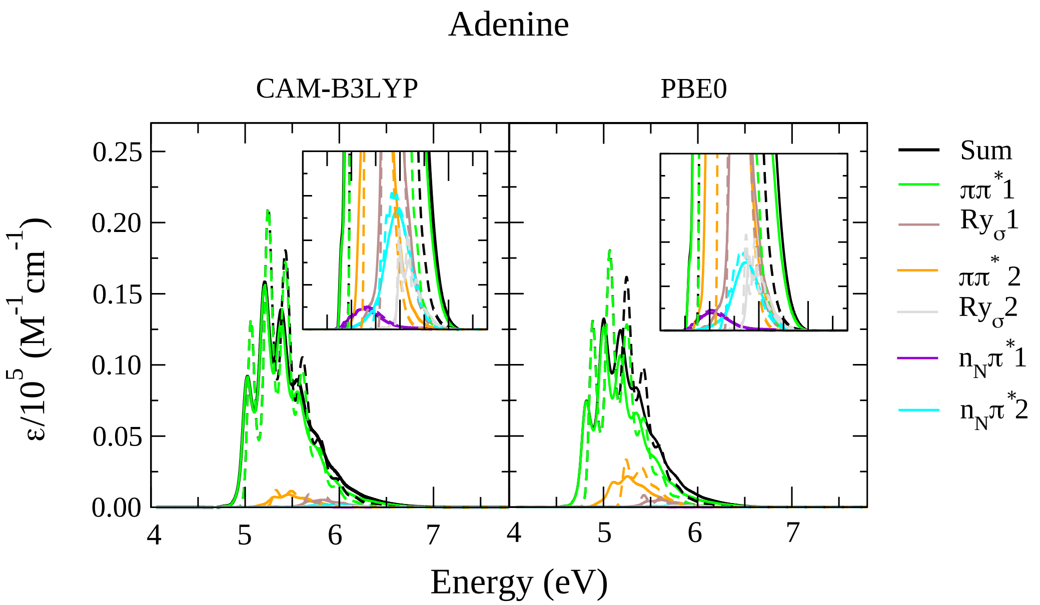

Figure S24: Absorption spectra of Adenine including individual state components from LVC models parametrized by CAM-B3LYP (left) and PBE0 (right) calculations, with 6-311+G(d,p) basis set and with (solid) and without (dashed) inter-state couplings.

S2.5 9H-Guanine

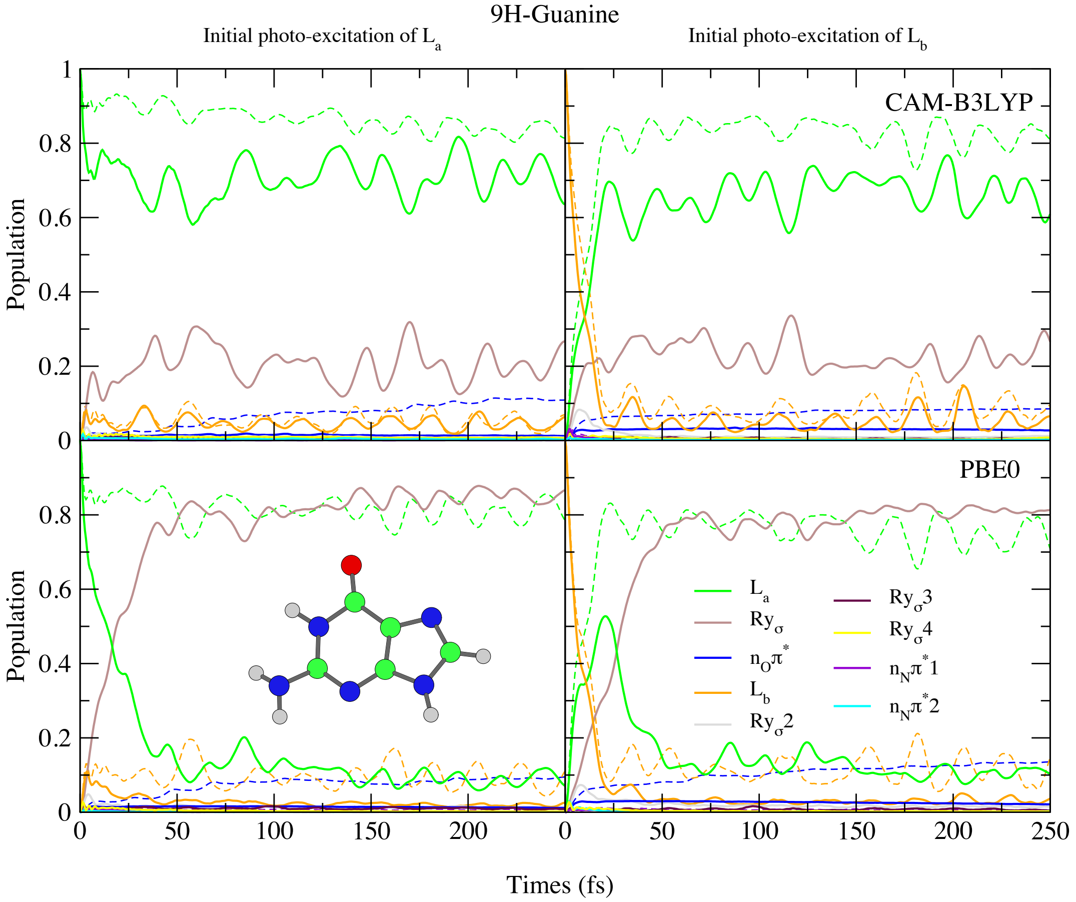

Figure S25: Nonadiabatic dynamics of electronic populations of 9H-Guanine in the gas phase, as predicted by an LVC Hamiltonian parameterized with calculations using CAM-B3LYP (top) and PBE0 (bottom) functionals with 6-31G(d) (dashed lines) and 6-311+G(d,p) (solid lines).

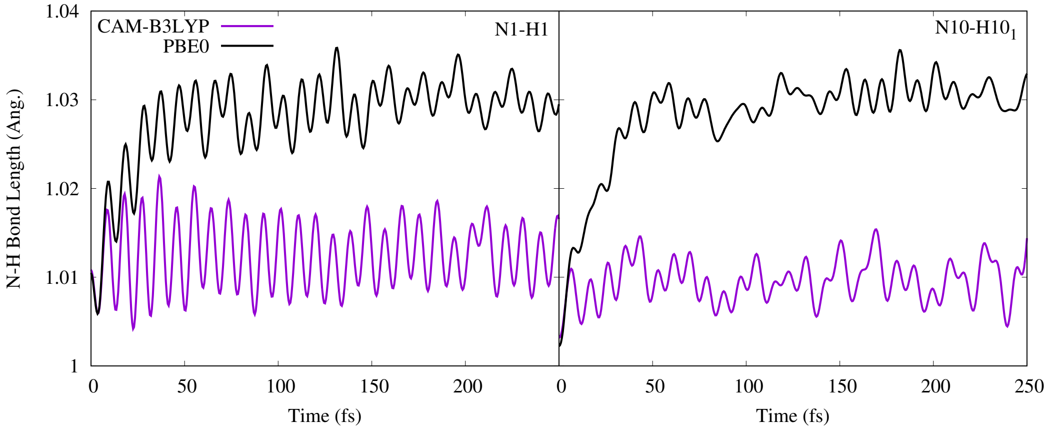

Figure S26: N1-H1 and N10-H10$_1$ bond lengths for CAM-B3LYP and PBE0 dynamics initiated on L_a state, calculated from the average position of the wavepacket during the dynamics.

Shown in Figure S26 is a small elongation of the N1-H1 and N10-H10$_1$ bonds during the dynamics calculations of 9H-Guanine where there is significant population on the lowest Rydberg state, particularly for the PBE0 model. The bond lengths of N1-H1 and N10-H10$_1$ in the minimum of the πRy$_\sigma$1 state in the LVC model for CAM-B3LYP are 1.025 Å and 1.024 Å versus 1.011 Å and 1.003 Å at the FC point. For PBE0 the bond lengths of N1-H1 and N10-H10$_1$ in the minimum of the πRy$_\sigma$1 in the LVC model are 1.030 Å and 1.032 Å versus 1.010 Å and 1.002 Å at the FC point.

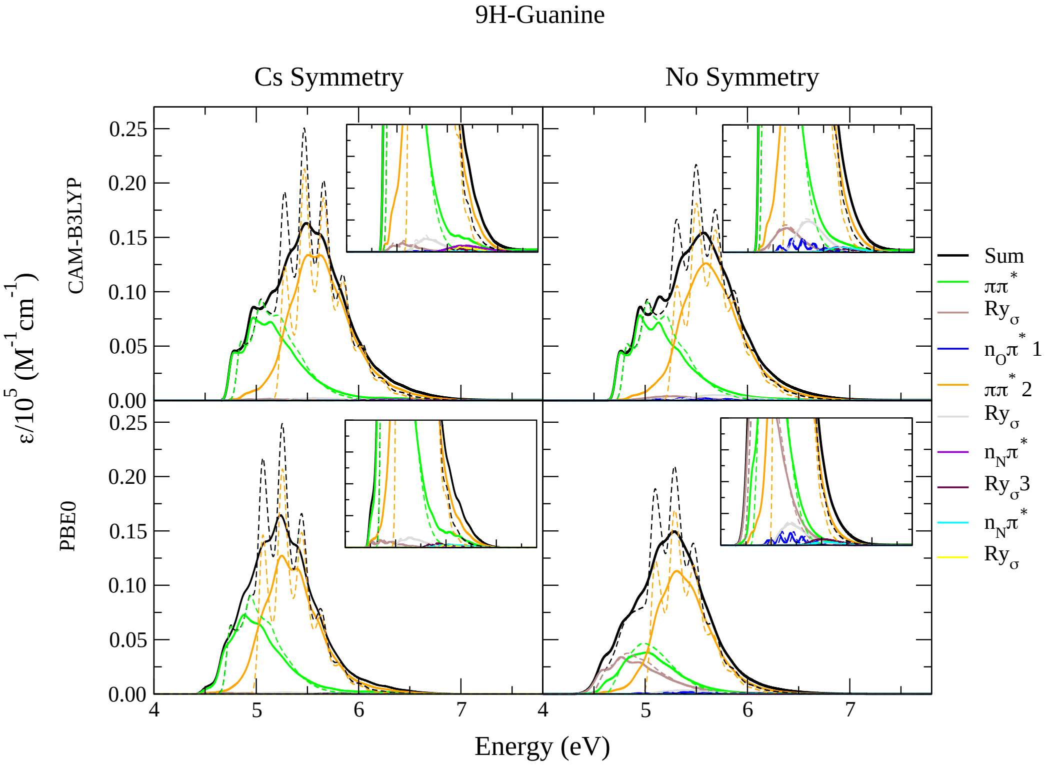

Figure S27: Absorption spectra of 9H-Guanine including individual state components from LVC models parametrized by CAM-B3LYP (top) and PBE0 (bottom) calculations, with 6-311+G(d,p) basis set, with (left) and without (right) C_s symmetry, and with (solid) and without (dashed) inter-state couplings.

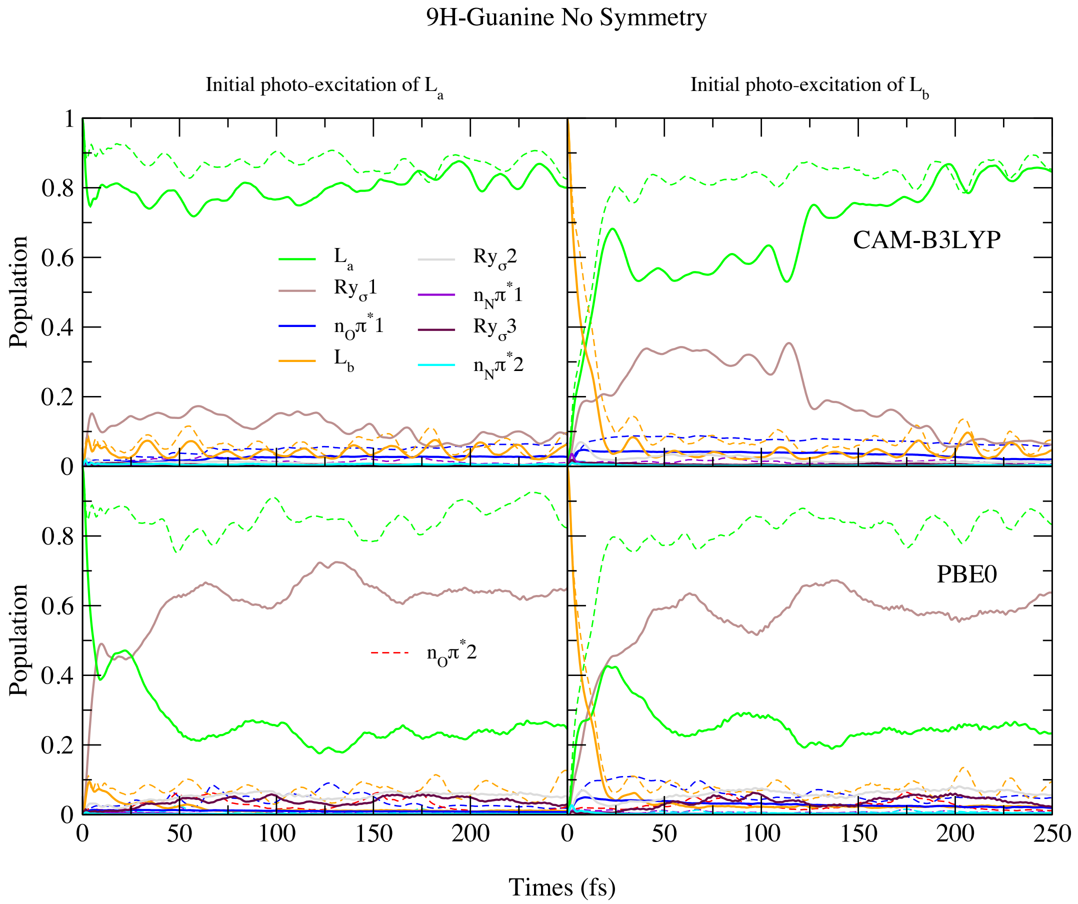

Figure S28: Nonadiabatic dynamics of electronic populations of 9H-Guanine without symmetry restrictions in the gas phase, as predicted by an LVC Hamiltonian parameterized with calculations using CAM-B3LYP (top) and PBE0 (bottom) functionals with 6-31G(d) (dashed lines) and 6-311+G(d,p) (solid lines). Only the PBE0(6-31G(d)) calculation includes the $n_O\pi^*2$ state, as for this level of theory it is found at a similar level to the $n_N\pi^*2$ state.

S2.6 7H-Guanine

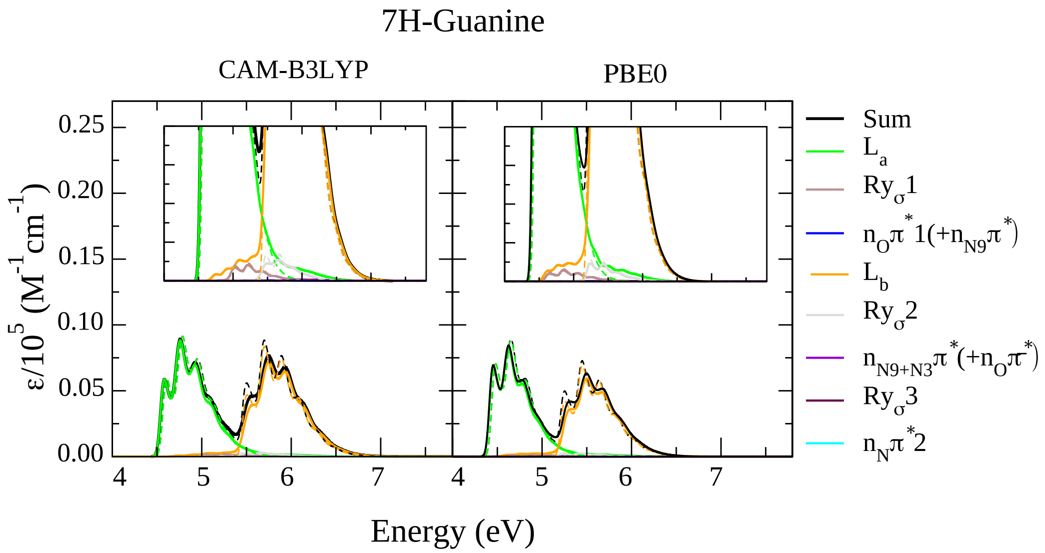

Figure S29: Absorption spectra of 7H-Guanine including individual state components from LVC models parametrized by CAM-B3LYP (left) and PBE0 (right) calculations, with 6-311+G(d,p) basis set and with (solid) and without (dashed) inter-state couplings.

References

[1] Melvin Lax, "The franck-condon principle and its application to crystals", *J. Chem. Phys.* **20**(11), pp. 1752–1760 (1952).

[2] Bao-Xin Xue, Mario Barbatti, and Pavlo O. Dral, "Machine learning for absorption cross sections", *J. Phys. Chem. A* **124**(35), pp. 7199–7210 (2020).
